# Magnetically separable and reusable rGO/Fe_3_O_4_ nanocomposites for the selective liquid phase oxidation of cyclohexene to 1,2-cyclohexane diol†

**DOI:** 10.1039/c9ra04685b

**Published:** 2019-10-11

**Authors:** Manoj Pudukudy, Qingming Jia, Yanan Dong, Zhongxiao Yue, Shaoyun Shan

**Affiliations:** Faculty of Chemical Engineering, Kunming University of Science and Technology Kunming 650500 Yunnan People's Republic of China manojpudukudy@gmail.com jiaqm411@163.com

## Abstract

A series of magnetically separable rGO/Fe_3_O_4_ nanocomposites with various amounts of graphene oxide were successfully prepared by a simple ultrasonication assisted precipitation combined with a solvothermal method and their catalytic activity was evaluated for the selective liquid phase oxidation of cyclohexene using hydrogen peroxide as a green oxidant. The prepared materials were characterized using XRD, FTIR, FESEM, TEM, HRTEM, BET/BJH, XPS and VSM analysis. The presence of well crystallized Fe_3_O_4_ as the active iron species was seen in the crystal studies of the nanocomposites. The electron microscopy analysis indicated the fine surface dispersion of spherical Fe_3_O_4_ nanoparticles on the thin surface layers of partially-reduced graphene oxide (rGO) nanosheets. The decoration of Fe_3_O_4_ nanospheres on thin rGO layers was clearly observable in all of the nanocomposites. The XPS analysis was performed to evaluate the chemical states of the elements present in the samples. The surface area of the nanocomposites was increased significantly by increasing the amount of GO and the pore structures were effectively tuned by the amount of rGO in the nanocomposites. The magnetic saturation values of the nanocomposites were found to be sufficient for their efficient magnetic separation. The catalytic activity results show that the cyclohexene conversion reached 75.3% with a highest 1,2-cyclohexane diol selectivity of 81% over 5% rGO incorporated nanocomposite using H_2_O_2_ as the oxidant and acetonitrile as the solvent at 70 °C for 6 h. The reaction conditions were further optimized by changing the variables and a possible reaction mechanism was proposed. The enhanced catalytic activity of the nanocomposites for cyclohexene oxidation could be attributed to the fast accomplishment of the Fe^2+^/Fe^3+^ redox cycle in the composites due the sacrificial role of rGO and its synergistic effect with Fe_3_O_4_, originating from the conjugated network of π-electrons in its surface structure. The rapid and easy separation of the magnetic nanocomposites from the reaction mixture using an external magnet makes the present catalysts highly efficient for the reaction. Moreover, the catalyst retained its activity for five repeated runs without any drastic drop in the reactant conversion and product selectivity.

## Abstract 2

A series of magnetically-separable and reusable rGO/Fe_3_O_4_ nanocomposites were successfully synthesized for the selective liquid-phase oxidation of cyclohexene to 1,2-cyclohexane-diol.

## Graphical abstract

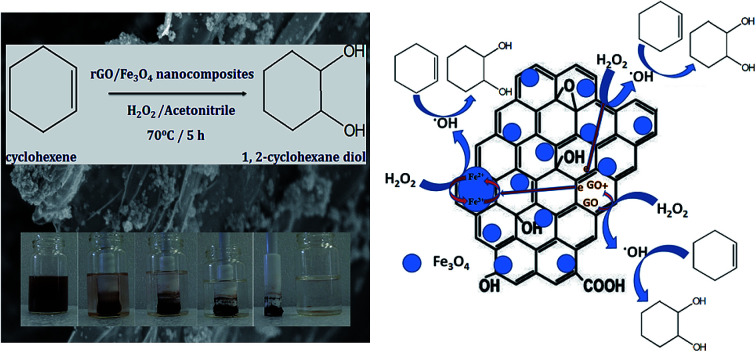

## 1. Introduction

In recent years, surface catalysis has become a hot area of research in synthetic chemistry because of its significance in organic transformation to produce high value added chemicals for various industries. Cyclohexene is one of the most widely used raw materials in chemical industries because of its low cost, availability and chemical structure.^1^ Since there are two potential oxidation sites existing in its structure, a mixture of different oxidation products was formed in its oxidation.^2^ Cyclohexene oxide (epoxide) and 1,2-cyclohexane diol (diol) are formed if the oxidation occurs at the double bond (C=C) position. However, if the oxidation occurs at the allylic position, then it leads to the formation of 2-cyclohexenol (enol) and 2-cyclohexenone (enone).^3^ All of these chemicals have promising use in various fields such as organic synthesis, materials science and pharmaceuticals.^4^ Therefore, the selective oxidation of cyclohexene to achieve any of these products is highly significant. Among the different oxidation products, diols have a significant role in the synthesis of pharmaceutical intermediates and it can be used as a direct precursor for the synthesis of adipic acid.^5^ In addition to this, it has a significant role in the synthesis of liquid crystals, resins and catechol. In the synthesis of epoxy resins, it is used as a diluter. Moreover, it acts as a chiral auxiliary in the asymmetric synthesis.^6^ Therefore, the selective synthesis of diol is highly important in pharmaceutical and chemical industries.

The common methods for diol synthesis are direct hydrolysis of epoxide and the dihydroxylation of cyclohexene. Dihydroxylation is the most widely accepted method and it uses the tetroxides of Os and Ru to catalyze the process, which acts as a catalyst and oxidant in this process.^7^ Because of the high cost, toxicity and non-selective nature the process, the use of these oxidants is limited and it is not possible for an industrial application. Furthermore, the reaction is very difficult to control using these oxidants. Therefore, it is highly desirable to develop an ecofriendly catalytic path for cyclohexene conversion to diol using greener oxidants and more efficient low-cost catalysts.

In the past few years, different types of homogeneous and heterogeneous catalysts, oxidants and solvents have been used for cyclohexene conversion.^8^ However, the conversion and selectivity of the products in cyclohexene oxidation was found to be completely depended on the type of catalyst, co-catalyst, oxidant and solvents used in the reaction.^9^ The commonly used catalysts for the oxidation of cyclohexene consists of metals-free or metals incorporated organic catalysts such as organometallic compounds and metal–organic frameworks, bare metal oxides, metal oxide hybrids, supported metal oxides and so on.^10–12^ Homogeneous catalysts were reported to be more effective for the liquid phase oxidation reactions and highly suitable to investigate the reaction mechanism. However, due to the difficulties associated with their isolation from reaction mixture and high susceptibility to drastic reaction conditions, their recyclability is restricted. These disadvantages of the homogeneous catalysts could be overcome by the use of heterogeneous catalysts.^13^ The metal-based catalysts are usually more advantageous than organocatalysts in the liquid phase oxidation because of their high stability, ease of separation and recyclability.^14,15^ Different types of oxidants were also used to catalyze the oxidation of cyclohexene.^16^ The main oxidants used are alkyl hydroperoxides, hydrogen peroxide, molecular oxygen and air. Among these, air, oxygen and hydrogen peroxide are considered as the green oxidants as their by-products are not harmful to environment.^17^ Moreover, among various alkyl hydroperoxides, *tert*-butyl hydroperoxide (TBHP) is the mostly used oxidant for cyclohexene oxidation. It usually yields cyclohexene oxide and allylic oxidation products based on the type of catalysts.^18,19^ The use of molecular oxygen as an oxidant for the reaction to produce allylic oxidation products was also reported over a set of different metal-based catalysts.^20–22^ The H_2_O_2_ assisted cyclohexene oxidation to 1,2-diol in a catalytic route has been also reported over a set of homogeneous and heterogeneous catalysts.^23–37^

For example, Usui *et al.*^26^ reported a maximum diol yield of 98% in a solvent- and metal-free cyclohexene oxidation using H_2_O_2_, catalyzed by immobilized sulfonic acids. In another work, Rosatella *et al.*^27^ reported the application of Brønsted acids in the catalytic conversion of cyclohexene to diol. Among the various catalysts used the *para*-toluene sulfonic acid was found to be showing the highest diol yield of 98%. Theodorou *et al.*^28^ used 2,2,2-trifluoroacetophenone as an organocatalyst for the oxidation of cyclohexene to diol using H_2_O_2_ and nearly 96% yield was observed for the product. Yu *et al.*^29,30^ reported the use of organoselenium compounds for the selective oxidation of cyclohexene to diol using H_2_O_2_ as the oxidant. Approximately 96% yield was observed for 42 hours of reaction using (PhSe)_2_ as catalyst at room temperature, whereas the reaction was completed within 3–5 hours, when the reaction performed with [3,5-(CF_3_)_2_C_6_H_3_Se]_2_. Even though these catalysts presented high efficiency for the reaction, their homogeneous nature limits their recyclability. Hence the need of heterogeneous catalysts is highly substantial in this reaction.

Huang *et al.*^32^ reported the direct use of polyaniline for the selective oxidation of cyclohexene to diol and the results shows that a highest cyclohexene conversion of 92.63% with 64.22% diol selectivity was noticed for the emeraldine base polyaniline with the size of 10–20 nm. The activity of the polyaniline is due to its small size and redox states. Zhou *et al.*^33^ reported the solvent free oxidation of cyclohexene to diol using acidic polyaniline@halloysite nanotubes. Approximately 98% conversion with 99.5% diol selectivity was achieved over PANI@HA/1 M/2.04-HCl catalyst at the optimized reaction conditions of 0.02 g catalyst, 2.5 mL H_2_O_2_ at 70 °C for 24 h of reaction. As per them, the enhanced activity of the catalysts could be ascribed to the reversible redox reaction of PANI in the composite for H_2_O_2_ decomposition. Boudjema *et al.*^34^ reported the montmorillonite clay supported 11-molybdovanado-phosphoric acid catalyst for the H_2_O_2_ assisted catalytic oxidation of cyclohexene to diol. The cyclohexene conversion and diol yield were reported to be 81.5% and 77% respectively for a 3 h of reaction at 70 °C. In other work, Guzik *et al.*^35^ reported cyclohexene oxidation over FDU-1-supported Nb(Co) catalyst, using H_2_O_2_ as oxidant in acetonitrile. The results indicated that a maximum cyclohexene conversion and diol selectivity of 61% and 56% was observed over the catalyst respectively.

It is reported that the oxides and complexes of transition metals especially Fe, Cu, V, Os, Mn, Ru, *etc.* were effectively used for dihydroxylation reaction.^7,38^ Since iron is the cheap and second most abundant metal, more efforts were given to the development of iron-based catalysts.^39^ Magnetic Fe_3_O_4_ is the main type of heterogeneous catalyst in this category due to its magnetic recovery from the reaction mixture for recyclability.^40^ However, due to the sintering and aggregation of particles, their catalytic activity was limited for an efficient reaction. This can be avoided by anchoring the metal oxides on a support material. The anchoring of metal oxides on a support has several advantages like the improvement of their catalytic activity and stability together with ease of recovery and recyclability. Carbon nanomaterials with two dimensional sheets like structures such as graphene, graphene oxide and reduced graphene oxide are of great importance in materials chemistry due to their ability to disperse the metal oxides on their surface.^41,42^ Thus, the development of highly efficient and low-cost catalysts for cyclohexene conversion with high diol selectivity is worthy.

In this work, a series of magnetically separable and reusable rGO/Fe_3_O_4_ nanocomposites were successfully prepared by an ultrasonication assisted precipitation combining solvothermal method and effectively used for the selective oxidation of cyclohexene to 1,2-cyclohexane diol using H_2_O_2_ as a green oxidant in liquid phase for the first time. It is reported that compared with co-precipitation method, the solvothermal route provides a more controlled growth and size of Fe_3_O_4_ nanoparticles for an efficient dispersion on the surface of graphene oxide.^43^ Additionally, it reduces the surface groups of graphene oxide and reduced graphene oxide (rGO) could be formed. The amount of GO was varied from 1 wt% to 10 wt% in the nanocomposites. To the best of our knowledge, the use of rGO/Fe_3_O_4_ nanocomposites for the liquid phase oxidations is very rarely reported as many of the works were focused on the degradation of dyes by a Fenton-type reaction. Previously Balasubramanyan *et al.*^39^ reported the use of a series of GO/Fe_3_O_4_ nanocomposites for the liquid phase oxidation of cyclohexene. Herein, a complete physico-chemical and magnetic characterization of the as-prepared rGO/Fe_3_O_4_ nanocomposites were studied and discussed in detail. The effects of various reaction parameters on cyclohexene conversion and product selectivity were investigated and correlated with catalytic properties. A plausible reaction mechanism for the improved activity/selectivity for oxidation is also reported.

## 2. Experimental

### 2.1 Materials

All of the chemicals used in the present study were purchased from Aladdin, China. These chemicals were of analytical grade and were used as received without further purification.

### 2.2 Synthesis of graphite oxide

A previously reported modified Hummer's method was used to synthesize the graphite oxide.^44^ In a typical procedure, 1 g of graphite powder and 0.5 g of sodium nitrate were added into 25 ml of conc. sulfuric acid taken in a 250 ml beaker. The mixture was kept in an ice bath to keep the temperature below 10 °C with vigorous magnetic stirring. Next 3 g of potassium permanganate was gradually added to the suspension. The temperature of the mixture was maintained below 15 °C during the addition of KMnO_4_ for a period of 3 h. After the complete addition of KMnO_4_, the ice bath was removed and the mixture was kept in a water bath at 35 °C and again stirred for 2 hours. Then 150 ml of deionized water was added to the mixture followed by the addition of 20 ml of 30% H_2_O_2_. This is to ensure the removal of residual permanganate and manganese dioxide in the samples. The suspension was then centrifuged to get a paste of graphite oxide. It was then washed four times with 10% HCl and five times with deionized water to remove any impurities in it. The as-synthesized graphite oxide was then dried in a vacuum oven at 60 °C for 24 h.

### 2.3 Synthesis of rGO/Fe_3_O_4_ nanocomposites

An ultra-sonication assisted precipitation combining solvothermal method was used to prepare the rGO/Fe_3_O_4_ nanocomposites. The amount of graphite oxide was varied during synthesis, according to the nominated amount of graphene oxide (GO) in the nanocomposites. In the present study, different compositions of GO were used, namely, 1 wt%, 2.5 wt%, 5 wt%, 7.5 wt% and 10 wt%. Firstly, the required amount of graphite oxide was dispersed in 200 ml of ethanol–water mixture (100 ml each) in an ultrasonic bath for 4 h to obtain the suspension of exfoliated graphene oxide. Next, a solution of FeCl_2_·4H_2_O and FeCl_3_·6H_2_O in 1 : 2 mol ratio was prepared in 200 ml of deionized water and it was mixed with the prepared exfoliated GO suspension and sonicated for 30 minutes. To precipitate the divalent and trivalent iron ions in the solution, aqueous ammonia was added slowly to the suspension by dropwise until the pH of the solution turned to 11. The blackish suspension was then directly transferred into a 500 ml Teflon lined stainless steel autoclave, sealed and kept in an air driven oven at 120 °C for 8 h. After solvothermal treatment, the autoclave was allowed to cool naturally and the precipitates were collected by manual filtration, washed several times with distilled water followed by ethanol and dried in a vacuum oven at 80 °C for 6 h to obtain the rGO/Fe_3_O_4_ nanocomposites. For comparison, bare Fe_3_O_4_ was also prepared by the same method, without the addition of GO. The dispersed GO suspension was also treated in similar solvothermal condition for studying its catalytic properties and activities. All of the samples were stored in air tight plastic containers for catalytic studies. The samples were labelled as *x*% rGO/Fe_3_O_4_, where *x* stands for the nominated amount of GO (wt%) in the nanocomposites.

### 2.4 Materials characterization

The as-prepared nanocomposites were characterized by using powder X-ray diffraction (XRD), Fourier transform infrared spectroscopy (FTIR), field emission scanning electron microscopy (FESEM), transmission electron microscopy (TEM), high resolution transmission electron microscopy (HRTEM), nitrogen physisorption, X-ray photoelectron spectroscopy (XPS) and vibrating sample magnetometer (VSM) analyses. The XRD analysis was performed in a Bruker D8 Focus X-ray Diffractometer with Cu Kα radiation at a wavelength of 0.1540 nm in the scanning range of 5 to 80° with a scan rate of 0.025°. The Scherrer's equation was used to calculate the crystallite size of the iron oxide from the full width at half maximum value of the intense diffraction peak, at the 2*θ* value of 35.6°. A Bruker VERTEX 70 FTIR instrument was used to record the FTIR spectra of the samples using KBr pellet method. The spectra were recorded in the region of 400–4000 cm^−1^ in transmission mode, after a background scan with pelletized KBr. The FESEM images of the prepared samples for the determination of their surface morphology were performed in a ZEISS, MERLIN system at an accelerating voltage of 3 kV. The samples were placed on a carbon tape, coated with iridium and photographed. A TECNAI G^2^ TF30 S-Twin TEM instrument was utilized for internal morphology characterization of the nanocomposites, which was operated at an accelerating voltage of 300 kV. The fast Fourier transform (FFT) electron diffraction patterns were also obtained together with the TEM micrographs. A small amount of the sample was dispersed in absolute ethanol by ultrasonication and a drop of the solution was placed on a carbon coated copper grid, dried under dim IR light and directly used for TEM imaging.

The nitrogen physisorption characterization was carried out in a Micromeritics ASAP 2020 system at −196 °C. The samples were degassed in vacuum at 100 °C for 2 h before analysis. The specific surface area and pore parameters of the samples were measured by Brunauer–Emmett–Teller (BET) and Barrett–Joyner–Halenda (BJH) methods respectively. The desorption branch of nitrogen isotherms was used for the calculations. The XPS characterization of the representative samples was studied in an ESCALAB 250Xi, Thermo Fisher Scientific XPS system. The advantage 5.967 surface chemical analyzer software was used to process and analyze the results. The binding energies of the elements were internally calibrated using the binding energy of C 1s (284.6 eV) as a reference. The narrow scan spectra were curve fitted using Gaussian–Lorentz function. The magnetic measurements were conducted on a MPMS3 SQUID vibrating sample magnetometer (Quantum Design) at room temperature. The electron spin resonance (ESR) spectroscopic analysis was carried out in a JEOL-JES FA200 ESR spectroscope, using 5,5-dimethyl-1-pyrroline-*N*-oxide (DMPO) as a radical trapping agent.

### 2.5 Liquid phase oxidation of cyclohexene

The catalytic performance of the prepared samples was investigated for the liquid phase oxidation of cyclohexene. The reactions were carried out in a two necked round bottom flask connected to a water condenser. In a typical procedure, the weighed amount of catalyst (0.05 g) was dispersed in a mixture of 2 mmol cyclohexene dissolved in 5 ml of acetonitrile solvent. Next the RB flask was placed in an oil bath at 70 °C and 10 mmol of H_2_O_2_ was slowly added to the reaction mixture as an oxidant (30% H_2_O_2_ in water, dropwise addition). The reaction was performed for 5 h. A constant magnetic stirring was provided in the whole period of reaction, for the proper mixing of the reactants, solvent and catalyst. After reaction, the catalysts were separated from the reaction mixture using an external magnet, and the reaction products were treated with anhydrous sodium sulphate to remove any residual water and were analyzed by a gas chromatograph (Agilent) connected to a flame ionization detector. An Elite-1 series capillary column was used for the separation and identification of the reaction products. The reactions were triplicated to confirm the results. The conversion of cyclohexene and selectivity of the formed products were calculated using the following equations, from the calibrated results of the standard samples. Cyclohexene conversion (%) = (([CH]_0_ − [CH]_*t*_)/[CH]_0_) × 100, where [CH]_0_ and [CH]_*t*_ represents the initial and final concentration of cyclohexene in moles respectively. Products selectivity (%) = (moles of individual product/total moles of products) × 100. The reaction parameters were further varied to study their effect on the cyclohexene conversion and products selectivity.

## 3. Results and discussion

### 3.1 Characterization of the prepared materials

**Fig. 1:**
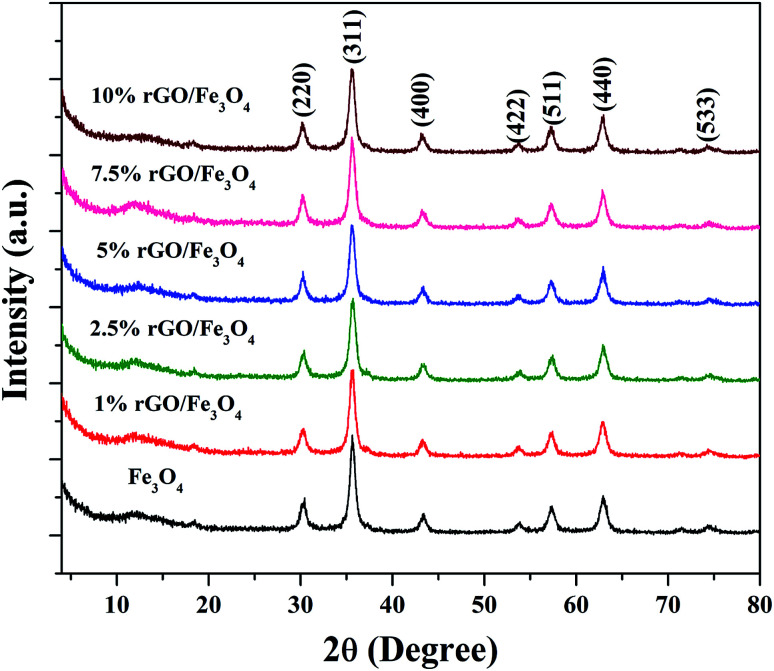
XRD patterns of the prepared samples.

The crystalline properties of the prepared samples were studied using XRD and the diffraction patterns are shown in Fig. 1. A sharp diffraction peak was observed at the 2*θ* value of 10.5° in Fig. S1.† This peak could be assigned to the (001) plane of the graphite oxide that has been synthesized by modified hummer's method. The interplanar distance (*d*) was measured to be 0.83 nm, which is in good agreement with the lamellar crystalline structure of graphite oxide.^45^ The other diffraction peak at the 2*θ* value of 20.3° might be initiated from the sample holder used for the XRD analysis. Fig. 1 shows the XRD patterns of the iron oxide and rGO/Fe_3_O_4_ nanocomposites. The diffraction peaks located at the 2*θ* values of 18.4°, 30.2°, 35.6°, 43.3°, 53.6°, 57.3°, 62.9° and 74.3° were directly indexed to the formation of cubic phase spinel crystalline structure of magnetite, Fe_3_O_4_, with the lattice constant of *a* = 8.375 Å (JCPDS card number: 00-065-0731). All of peaks of Fe_3_O_4_ were found to be completely retained in the prepared rGO/Fe_3_O_4_ nanocomposites. It indicates the successful anchoring of Fe_3_O_4_ on reduced graphene oxide matrix. However, no peaks related to graphene oxide or rGO were observed in the prepared nanocomposites. This could be due to the excellent dispersion of highly exfoliated reduced graphene oxide sheets or due to the low amount of GO in the nanocomposites. No other diffraction peaks were observed, which further points to the absence of impurities in the nanocomposites. The high intensity of the diffraction peaks in the nanocomposites suggests that the Fe_3_O_4_ particles are well crystallized. However, the incorporation of rGO contributed a small reduction in the intensity and a broadening of the diffraction peaks was detected in the composites. This could be attributed to the size-controlled growth of Fe_3_O_4_ nanoparticles on reduced graphene oxide. The incremental incorporation of graphene oxide from 1 wt% to 10 wt% does not altered the crystallinity of Fe_3_O_4_ in nanocomposites. The crystallite size of Fe_3_O_4_ calculated using Scherrer's equation was completely complemented with this observation. The average crystallite size of Fe_3_O_4_ was calculated to be 11 nm and 7 ± 2 nm in the bare and rGO incorporated Fe_3_O_4_ nanocomposites respectively. It is previously reported that the graphene oxide is a good support for nanoparticles as it provides necessary surface features through its functional groups.^46^ Therefore, the growth of particles could be effectively controlled without any severe particle agglomeration. A similar view is observed here in the case of the prepared nanocomposites.

**Fig. 2:**
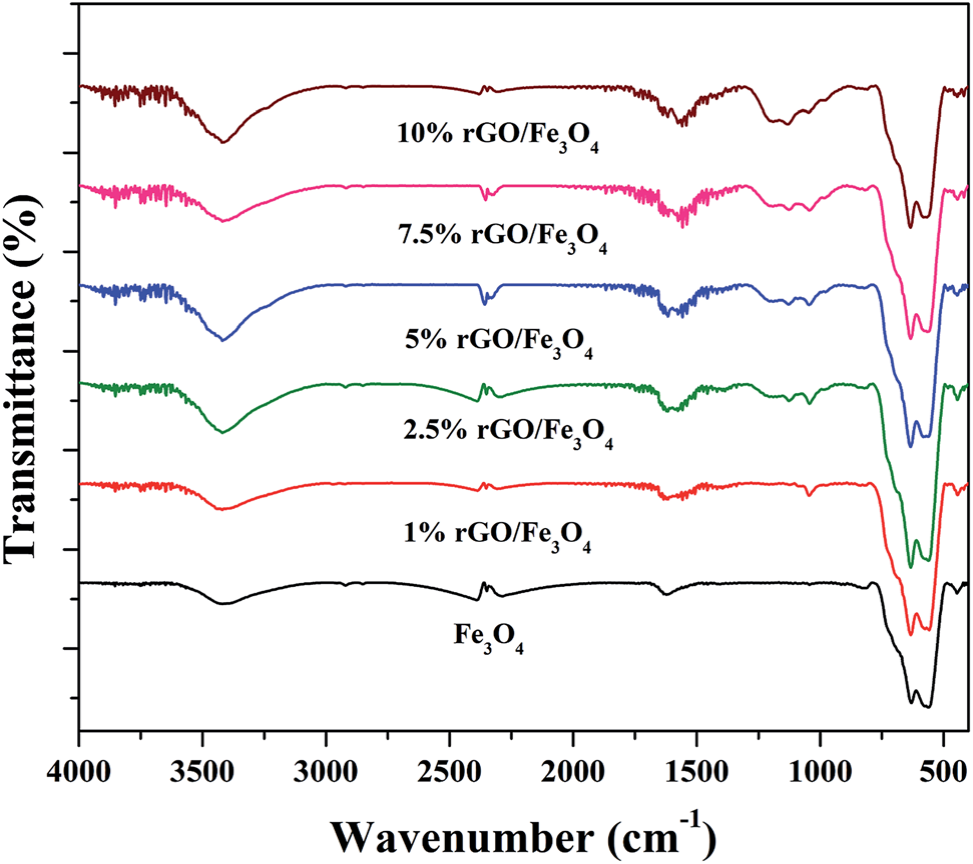
FTIR spectra of the prepared samples.

The surface chemistry and bonding characteristics of the prepared materials were studied using FTIR spectroscopy and the spectra are shown in Fig. 2. The broad and intense transmission band found in the region of 495–775 cm^−1^ and a less intense transmission band at 445 cm^−1^ were related to the stretching vibration of Fe–O bond.^47^ These two transmission bands confirm the presence of iron oxide, specifically Fe_3_O_4_ in the samples. This is highly consistent with the XRD results. The other transmission bands centered at 1619 cm^−1^ and 3423 cm^−1^ were attributed to the bending and stretching vibrations of the O–H group, originated from the adsorbed water molecules. These bands were found to be retained in the nanocomposites and their intensity increased with the increasing amount of graphene oxide in the composites. This could be attributed to the presence of more surface hydroxyl groups in the composites.^48^ Some other weakly resolved and less intense transmission bands were also identified in the nanocomposites with increasing amount of GO. It could be due to presence of oxygen containing functional groups in the composites arising from partially-reduced graphene oxide. The transmission band located at 1043 cm^−1^ could corresponds to the stretching vibration of C–O originated from the presence of oxygen on the surface of graphene oxide formed by the oxidation of graphite.^49^ The transmission band observed at 1226 cm^−1^ was related to the symmetric stretching vibration of the epoxy and C–OH groups in the nanocomposites. The transmission bands located at 1627 cm^−1^ and 1735 cm^−1^ denote the OH bending and C=O stretching vibration of the COOH group.^50^ The aromatic C=C bond and carbonyl bond also showing the same transmission bands in these region.^51^ The low intensity of the functional groups fully validates the reduction of surface groups of GO after solvothermal treatment. However, only a partial reduction of graphene oxide was resulted in the nanocomposites. It could be ascribed to the applied conditions of the solvothermal treatment.

**Fig. 3:**
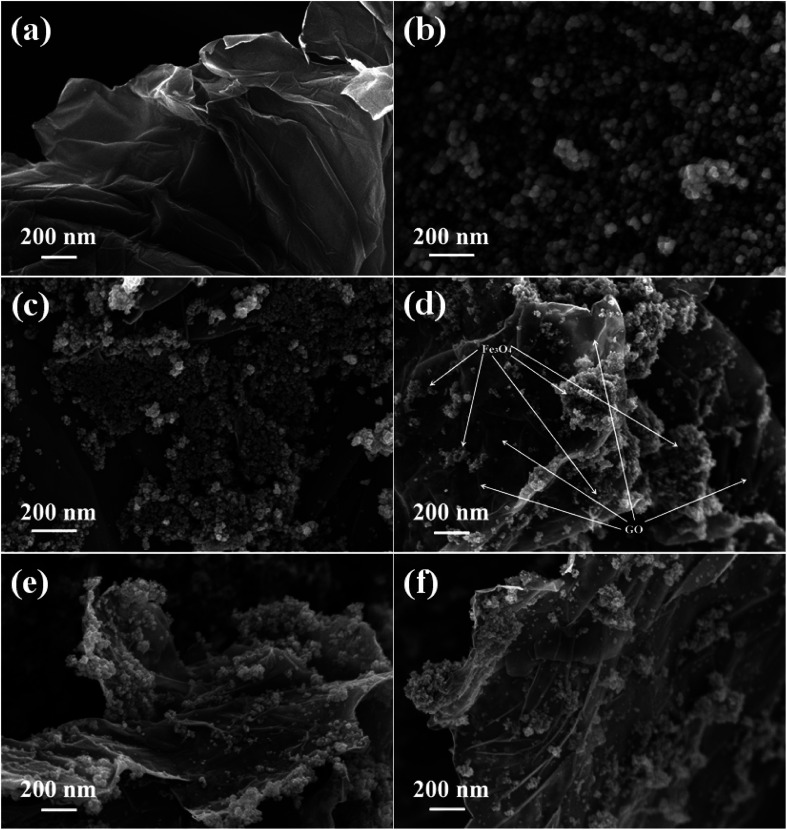
FESEM images of the as-synthesized samples (a) GO, (b) Fe_3_O_4_, (c) 1% rGO/Fe_3_O_4_, (d) 2.5% rGO/Fe_3_O_4_, (e) 5% rGO/Fe_3_O_4_ and (f) 7.5% rGO/Fe_3_O_4_ nanocomposites.

The morphological features of the prepared samples were studied using FESEM analysis and the images are shown in Fig. 3. The sheet like morphology of the graphite oxide is clearly shown in Fig. 3(a), whether the surface layers of graphene oxide are clearly visible. A number of wrinkles and ripples were also seen on its puffy surface. A cluster of more or less spherical shaped Fe_3_O_4_ particles with a moderate degree of particle aggregation is shown in Fig. 3(b). The size of the Fe_3_O_4_ particles was found to be uniform and it was measured to be in the nano range of 10–12 nm. The introduction of graphene oxide allowed the homogeneous dispersion of Fe_3_O_4_ nanoparticles on the surface of reduced graphene oxide in the nanocomposites.

As shown in Fig. 3(c), the Fe_3_O_4_ nanoparticles were uniformly distributed over reduced graphene oxide sheets in the 1% rGO/Fe_3_O_4_ nanocomposite. However, due to its low amount, the individual layered structure of reduced graphene oxide was not seen. It is expected that the rGO surfaces were completely covered with Fe_3_O_4_ nanoparticles. When the amount of graphene oxide increased to 2.5 wt% and more, the layers of reduced graphene oxide sheets were clearly visible in the composites as shown in Fig. 3(d)–(f). The Fe_3_O_4_ nanoparticles were found to be finely dispersed and anchored on the surface layers of reduced graphene oxide. The rate of growth and aggregation of particles is found to be fully controlled by the incorporation of graphene oxide in the nanocomposites. The surface edges of reduced graphene oxide layers were also found to be fully decorated with spherical shaped iron oxide nanoparticles as seen in the SEM images of 2.5% to 7.5% rGO combined composites. The presence of oxygenated surface functional groups in graphene oxide such as hydroxyl, epoxy and carboxyl groups could enable the homogeneous anchoring and decoration of iron oxide nanoparticles on the layered surfaces of the reduced graphene oxide with a tight bond between the individual components of the nanocomposites.^52^ Moreover, the two-dimensional layered nanostructure of reduced graphene oxide was not affected by the loading of Fe_3_O_4_ nanoparticles and its structure was preserved in all of the nanocomposites. The ultra-dispersion of Fe_3_O_4_ nanoparticles on rGO surface restricts the particle growth and hence large Fe_3_O_4_ particles and bigger aggregates of Fe_3_O_4_ clusters were not seen in the prepared nanocomposites. The size of Fe_3_O_4_ nanoparticles was measured to be approximately 6–8 nm in all of the nanocomposites, which is smaller than that observed in the case of bare Fe_3_O_4_. Moreover, the aggregation of Fe_3_O_4_ nanoparticles was found to be decreased with increasing the amount of graphene oxide in the nanocomposites. It could be due to the availability of sufficient rGO surface for an effective dispersion of Fe_3_O_4_ nanoparticles without any severe aggregation of particles.

**Fig. 4:**
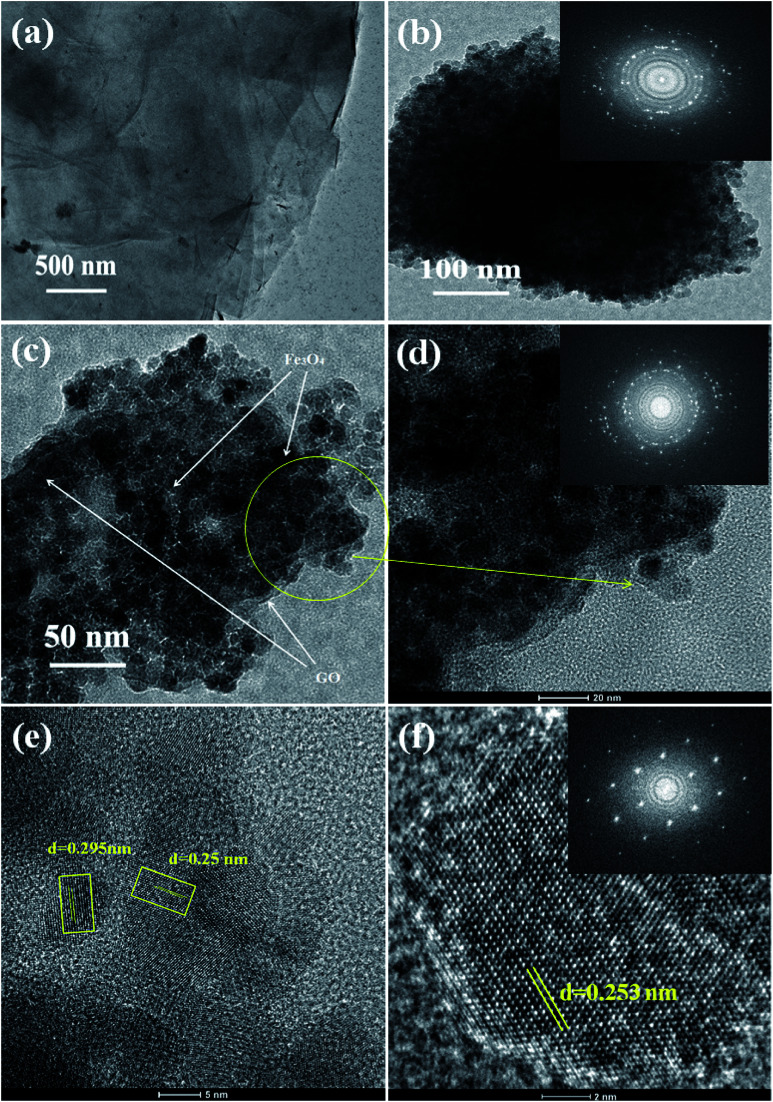
TEM images of the prepared samples (a) rGO, (b) Fe_3_O_4_ (c, d) 5% rGO/Fe_3_O_4_ nanocomposite and (e, f) high resolution TEM images of the representative composite (FFT patterns were shown in the inset).

The internal structure and degree of particle dispersion in the prepared samples were evaluated by TEM analysis and the resulting images are shown in Fig. 4. The transparent layered surface structure of the rGO sheets and a cluster of aggregated Fe_3_O_4_ nanoparticles prepared without graphene oxide are shown in Fig. 4(a) and (b) respectively. The electron diffraction pattern shown in the inset of Fig. 4(b) indicates the polycrystalline nature of the Fe_3_O_4_ nanoclusters. The presence of Fe_3_O_4_ nanoparticles decorated on the surface of exfoliated reduced graphene oxide layers was displayed in the TEM image of 5% rGO/Fe_3_O_4_ nanocomposite. The rGO layers and Fe_3_O_4_ nanoparticles were clearly marked in the TEM photograph of the representative nanocomposite, as shown in Fig. 4(c). The size of more or less spherical shaped Fe_3_O_4_ nanoparticles distributed on rGO layers was measured to be varying from 5–8 nm, which is in good agreement with the XRD and SEM observations.

The inset figure shown in the high magnified TEM image of the 5% rGO/Fe_3_O_4_ nanocomposite in Fig. 4(d) comprises well developed concentric diffraction rings with sharp diffraction spots. It indicates the polycrystalline nature of Fe_3_O_4_ in the composites. The high resolution TEM image showed in Fig. 4(e) displays clear lattice fringes of iron oxide. The lattice fringe spacings were measured to be 0.295 nm and 0.25 nm, which corresponded to the (220) and (311) reflections of the Fe_3_O_4_ respectively. Fig. 4(f) represents the HRTEM image and fast Fourier transform pattern of a single crystalline Fe_3_O_4_ particle in the nanocomposite. The particle was found to have a size of ∼8 nm and a lattice spacing of 0.253 nm, which is consistent with its (311) plane.

**Fig. 5:**
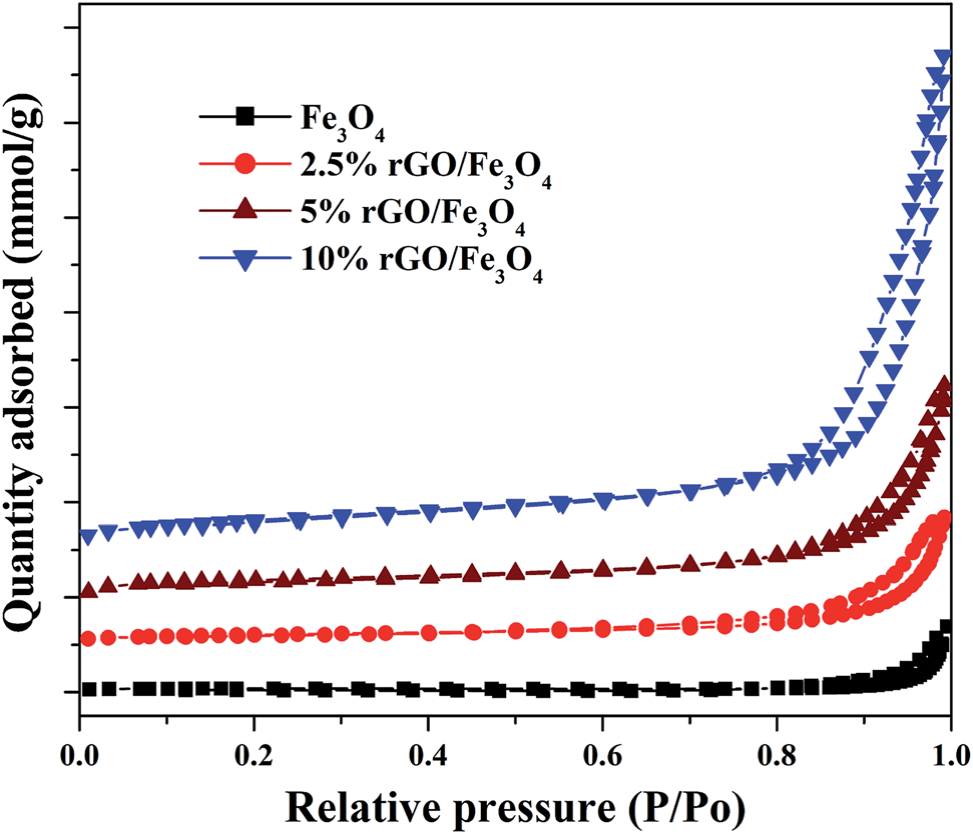
Nitrogen sorption isotherms of the prepared samples.

The textural properties of the Fe_3_O_4_ and rGO/Fe_3_O_4_ nanocomposites were studied using BET/BJH analysis and the nitrogen physisorption isotherms are shown in Fig. 5. According to IUPAC classification, the samples show isotherms that belong to type IV, with a H1 hysteresis loop. It indicates the presence of mesopores in the prepared samples.^53^

The hysteresis loops were observed to be widened by the incorporation of a small amount of graphene oxide and the trend was continued with its increasing amount in the nanocomposites. The specific surface area of Fe_3_O_4_ nanoparticles was measured to be 56.37 m^2^ g^−1^ by the BET method. However, the incorporation of 2.5% GO, increased the surface area to 106.28 m^2^ g^−1^, *i.e.* the surface area of Fe_3_O_4_ was almost doubled. This could be due to the efficient dispersion of Fe_3_O_4_ nanoparticles over rGO without particle agglomeration. The surface area of graphite oxide prepared in this work was measured to be 738.34 m^2^ g^−1^. The incremental addition of graphene oxide further increased the surface area of the nanocomposites significantly due to the synergistic effect of Fe_3_O_4_ nanoparticles on the surface layers of rGO. The surface area was measured to be 138.15 m^2^ g^−1^ and 239.67 m^2^ g^−1^ for the 5% and 10% GO incorporated nanocomposites respectively. The high surface area of the nanocomposites further confirms that the graphene oxide is a good support for the effective immobilization of Fe_3_O_4_ nanoparticles on its surface. Similar results were previously reported by other researchers.^54^ The average pore size and total pore volume of the samples were measured using BJH method and was found to be 4.8 nm, 16.6 nm 18.6 nm and 20.1 nm and 0.175 cm^3^ g^−1^, 0.219 cm^3^ g^−1^, 0.258 cm^3^ g^−1^ and 0.328 cm^3^ g^−1^ for the bare Fe_3_O_4_, 2.5%, 5% and 10% rGO incorporated composites respectively. Like surface area, the pore parameters of Fe_3_O_4_ were also found to be increased by the incorporation of graphene oxide and its amount in the nanocomposites. The BJH plots of the nanocomposites indicated the presence of different types of pores in them, whereas only one kind of pore with the size of 4.8 nm was noted for the bare Fe_3_O_4_ and which could be originated from the inter-aggregation of Fe_3_O_4_ nanoparticles. It further indicates the enhanced role of GO in tailoring the pore structures of the nanocomposites.^55^ Moreover, the increased pore volume of the nanocomposites could be due to the formation of secondary pores as discussed before, which may originate from the close stacking between the rGO and Fe_3_O_4_ nanoparticles in the composites as reported.^56^ The large surface area and pore volume of the nano-composites makes more catalytic active sites for a catalytic reaction and it would be better for a facile mass diffusion and transmission, providing improved catalytic activity.

**Fig. 6:**
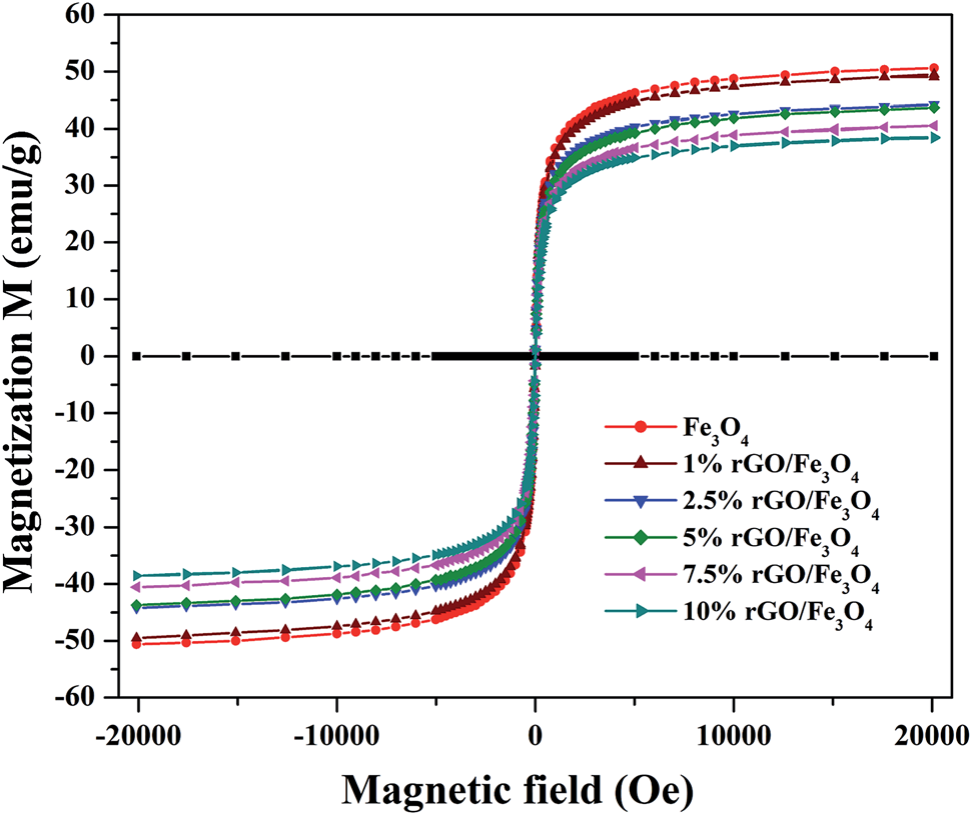
Magnetic hysteresis loops of the prepared samples.

The magnetic properties of the prepared samples were studied using VSM analysis and the magnetization curves of the bare Fe_3_O_4_ and rGO/Fe_3_O_4_ nanocomposites were tested in the magnetic field of ±20 kOe at room temperature. The resulting M–H curves are shown in Fig. 6. The S-shaped magnetization hysteresis loops indicates the superparamagnetic nature of the prepared samples with immeasurable remanence and coercivity. The saturation magnetization value of Fe_3_O_4_ nanoparticles was measured to be 51.2 emu g^−1^, which is significantly lower than the theoretical magnetization value of 92 emu g^−1^ for the bulk Fe_3_O_4_. This could be due to the effects of surface contribution and non-stoichiometry in the prepared sample.^57^ The surface contribution could be mainly from the pinning of the surface spins at the particle interface. However, this value is very close to the most of the reported values of Fe_3_O_4_ nanoparticles.^58^ The saturation magnetization values of the rGO incorporated nanocomposites were found to be 49.3 emu g^−1^, 44.3 emu g^−1^, 42.9 emu g^−1^, 40.5 emu g^−1^ and 38.02 emu g^−1^ respectively for the 1%, 2.5%, 5%, 7.5% and 10% graphene oxide incorporated nanocomposites. It is clear that the saturation magnetization value of Fe_3_O_4_ was slightly decreased to 49.3 emu g^−1^ with the incorporation of 1% rGO. It was further decreased to 38.02 emu g^−1^ for the 10% nanocomposite. The decrease in the saturation magnetization could be ascribed to the low quantity of magnetic Fe_3_O_4_ and the presence of non-magnetic graphene part in the nanocomposites. Furthermore, the strong anchoring of highly dispersed Fe_3_O_4_ nanoparticles on the surface rGO layers reduces the magnetization moment.^59^ It additionally confirms the formation of composite structure. However, the low saturation magnetization values of the composites are still more than enough to ensure its efficient magnetic separation. The superparamagnetic behavior and the non-significant coercivity and remanence make the easy and quick magnetic recovery of the as-prepared nanocomposites from the solution by applying an external magnetic field. The removal of magnetic field again allows its dispersion in solution. Fig. S2† shows the effective magnetic recovery of the nanocomposite from reaction mixture.

**Fig. 7:**
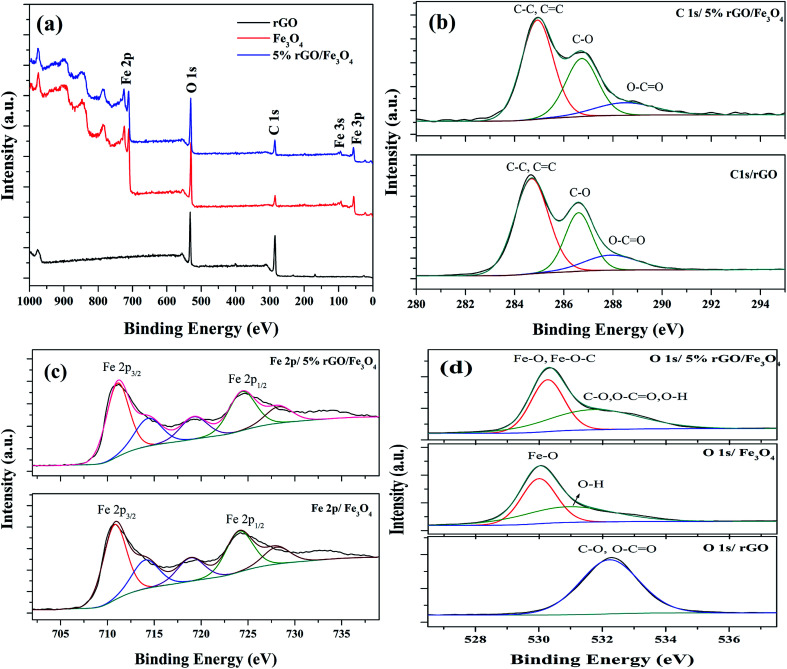
XPS spectra of the prepared samples (a) survey scan spectra and curve fitted narrow scan spectra of (b) C 1s, (c) Fe 2p and (d) O 1s of the rGO, Fe_3_O_4_ and 5% rGO/Fe_3_O_4_ nanocomposites.

The chemical composition and surface states of the elements present in the prepared samples were investigated using XPS analysis and the wide scan survey spectra of rGO, Fe_3_O_4_ and 5% rGO/Fe_3_O_4_ nanocomposite are shown in Fig. 7(a). As shown, the rGO contained C and O as the elements at the binding energies of 284.6 eV and 531.1 eV respectively. The Fe_3_O_4_ and 5% rGO/Fe_3_O_4_ nanocomposite contained C, O and Fe as the elements at the same binding energy positions of 284.6 eV, 531.1 eV and 710.5 eV respectively.^60^ The absence of other elemental peaks confirms the high purity of the prepared samples. The peak for C in the Fe_3_O_4_ sample could be originated from the carbon used for XPS analysis, which cannot be discarded. The presence of rGO in the composite was correctly identified by comparing the peak intensities of C 1s. As seen, the peak intensity of C 1s in the composite was higher than that of the same in bare Fe_3_O_4_, confirming the formation of nanocomposites. The curve fitted narrow scan XPS spectra of C 1s are shown Fig. 7(b). As shown in the C 1s of rGO, the peak at the binding energy of 284.6 eV was assigned to the sp^3^ and sp^2^ carbon species of the reduced graphene oxide such as C–C and C=C bonding.^61^ The peaks at higher binding energies were assigned to the oxygenated surface groups in graphene oxide. The peak at 286.6 could be related to the C–O bonding whereas the peak at 288.0 eV could be assigned to the C=O bonds.^62^ The high intensity of the peak at 284.6 eV (C–C/C=C) compared to the other two peaks at 286.6 eV and 288 eV with low intensities confirm the reduction of graphene oxide by the solvothermal treatment. The same peaks were found to be retained in the C 1s spectrum of the nanocomposite. However, the peak positions were slightly shifted to higher binding energies. The binding energies of the peaks related to C–C/C=C, C–O, C=O bondings were measured to be 284.95 eV, 286.72 eV and 288.66 eV respectively. The shift of the binding energies of C 1s peaks in the rGO/Fe_3_O_4_ nanocomposite is not surprising since similar observations were previously reported.^63^ It could be ascribed to the interaction of these groups with Fe_3_O_4_ nanoparticles anchored on the surface of rGO.

Fig. 7(c) shows the deconvoluted narrow scan spectra of Fe 2p. There are two main peaks were observed at the binding energies of 710.8 eV and 724.1 eV in the Fe 2p spectra of bare Fe_3_O_4_. These values were attributed to the Fe 2p_3/2_ and Fe 2p_1/2_ spin orbit peaks of Fe_3_O_4_ respectively.^64^ The other deconvoluted peaks at the binding energies of 714.14 eV, 718.79 eV and 728.16 eV could correspond to their satellite peaks. These values were highly consistent with the standard binding energies of Fe_3_O_4_.^65^ As observed in the case of C 1s, the positions of Fe 2p peaks in the composite were also shifted to higher binding energies. The binding energies of Fe 2p_3/2_ and Fe 2p_1/2_ were found to be 711.17 eV and 724.66 eV, due to their strong interaction with rGO through Fe–O–C bonds. Their satellite peaks also shadowed the same shift to higher binding energies. The curve fitted O 1s spectra of all of the samples were shown in Fig. 7(d). Only one peak at the binding energy of 532.1 eV was observed in the O 1s of reduced graphene oxide. This peak could be assigned to the presence of partially reduced oxygen containing groups such as C–O, O–C=O and O–H groups in it.^66^ The deconvoluted O 1s spectra of Fe_3_O_4_ showed two peaks at the binding energies of 530 eV and 531.1 eV. The peak at 530 eV corresponded to the lattice oxygen of Fe_3_O_4_, whereas the peak at 531.1 eV was related to the surface adsorbed hydroxyl groups. The O 1s spectra of the composite also showed two deconvoluted peaks at the binding energies of 530.3 eV and 531.98 eV. The peak due to lattice oxygen was found to be quite shifted to higher binding energies compared to that of bare Fe_3_O_4_. This could be attributed to their interaction with reduced graphene oxide *via* Fe–O–C bondings.^67^ However, the broad peak at 531.98 eV could be related to the presence of oxygen containing groups such as C–O, O–C=O and O–H groups in the composites, left after the partial reduction of GO in the solvothermal reaction.

### 3.2 Catalytic activity of the rGO/Fe_3_O_4_ nanocomposites

The catalytic activity of the as-prepared nanocomposites was investigated for the liquid phase oxidation of cyclohexene using H_2_O_2_ as a green oxidant. The main products obtained in the present study were identified to be 1,2-cyclohexane diol, cyclohexene oxide, 2-cyclohexenol and 2-cyclohexenone as discussed in the introduction. However, high selectivity was observed for the diol product over rGO/Fe_3_O_4_ nanocomposites using H_2_O_2_. Some preliminary experiments were first conducted to study the effect of catalytic reaction.

**Fig. 8:**
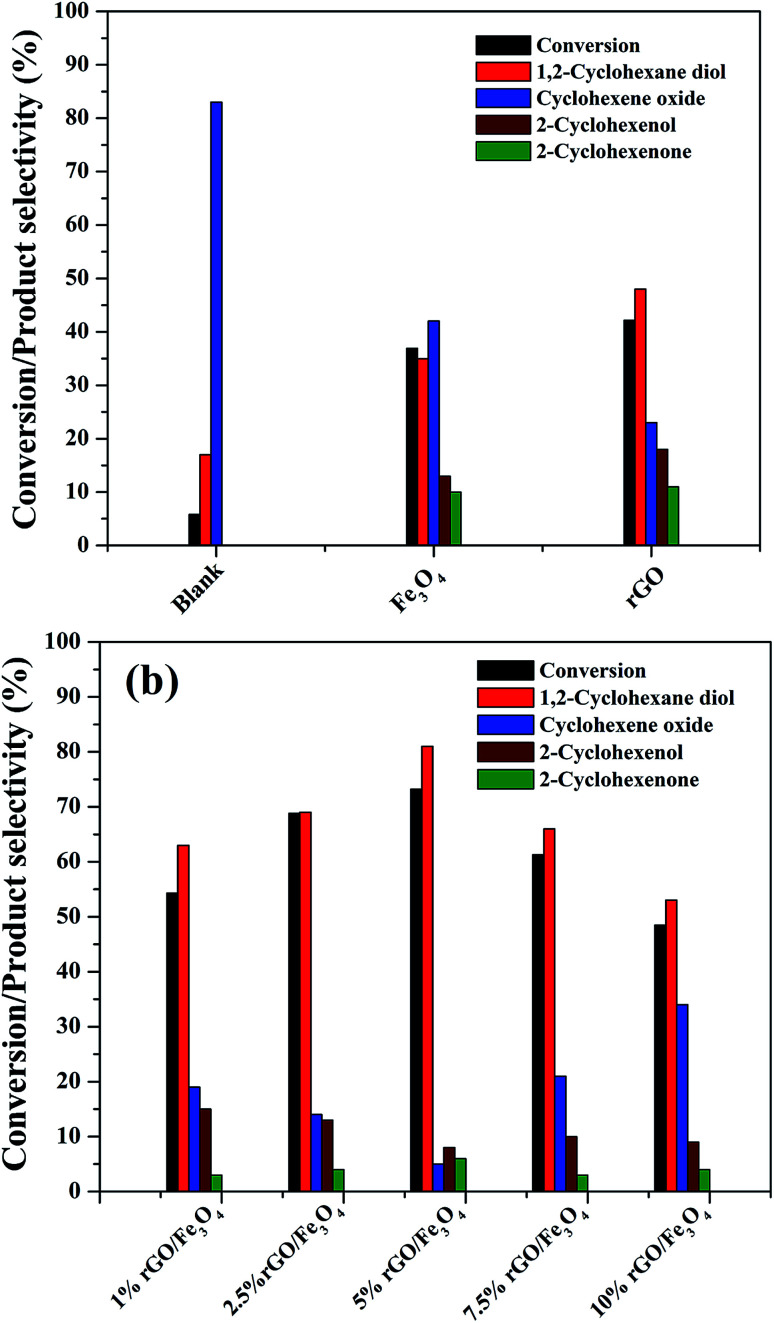
Catalytic performance of the as-synthesized samples for the liquid phase oxidation of cyclohexene (a) preliminary experiments (b) reactions over rGO/Fe_3_O_4_ nanocomposites with various amounts of GO (*cyclohexene: 2 mmol, H*_*2*_*O*_*2*_*: 10 mmol, acetonitrile: 5 ml, catalyst dosage: 0.05 g, temperature: 70 °C, reaction time: 5 hours, atmospheric pressure*).

As seen in Fig. 8(a), a maximum cyclohexene conversion of 5.8% with 83% epoxide selectivity was reached in absence of catalysts for 5 h of reaction. When bare Fe_3_O_4_ and rGO was used as a catalyst, the conversion reached to 36.9% and 42.2% with a diol selectivity of 35% and 48% respectively. The low activity of the bare Fe_3_O_4_ could be attributed to the aggregation Fe_3_O_4_ nanoparticles. The activity of the rGO could be due to its enhanced self-sacrificial donor–accepter character due to the presence of sp^2^ and sp^3^ carbon atoms together with the presence of unreduced surface acidic functional groups present in it.^67,68^ However, cyclohexene oxide was also formed in high proportions with the selectivity of 42% and 23% respectively. In both cases the formation of allylic oxidation products such as cyclohexenol and cyclohexenone were observed, which dropped the overall selectivity of the products. In the blank run no allylic oxidation products were obtained.

Fig. 8(b) shows the effect of different amount of rGO in the rGO/Fe_3_O_4_ nanocomposites for cyclohexene oxidation. It can be clearly seen that the introduction of 1% rGO onto Fe_3_O_4_ increased the cyclohexene conversion to 53.3% with a diol selectivity of 63%. It indicates the enhanced role of rGO/Fe_3_O_4_ nanocomposites for the selective liquid phase oxidation of cyclohexene using H_2_O_2_ as oxidant. With increasing the amount of rGO, the conversion and diol selectivity increased significantly until an optimum value of rGO and thereafter a drastic drop was noticed in the catalytic activity. Using 2.5% rGO/Fe_3_O_4_, the conversion increased to 68.8% with 69% selectivity for the diol product. However, a maximum cyclohexene conversion of 73.2% with a highest diol selectivity of 81% was obtained over 5% rGO/Fe_3_O_4_ nanocomposite, which is the highest value among the prepared samples for 5 h of reaction. The further increase of graphene oxide in the composite resulted in a low conversion of cyclohexene and a low selectivity for diol formation. Over 7.5% and 10% rGO incorporated composites, the cyclohexene conversion and diol selectivity were calculated to be 61.3% and 48.5% and 66% and 53% respectively.

From these results it is seen that the amount of rGO is a crucial factor for the activity of the nanocomposite for cyclohexene oxidation. Even though a low amount of rGO (1%) makes the Fe_3_O_4_ more effective, it is not enough to achieve a good conversion and selectivity of the product. This could be probably connected to the dispersion rate of Fe_3_O_4_ nanoparticles on the surface of rGO along with their surface area. The low amount of GO might not be sufficient for the dispersion of Fe_3_O_4_ nanoparticles effectively on the surface of rGO without particle aggregation. For an effective catalytic reaction, the active metal dispersion is highly important, and it is expected that the 5% rGO provides a better dispersion for Fe_3_O_4_ nanoparticles compared to 1% and 2.5% rGO incorporated nanocomposites. This makes the 5% rGO/Fe_3_O_4_ nanocomposite more effective for the cyclohexene oxidation reaction. However, the decreased performance of the nanocomposites with high rGO content such as 7.5% and 10% could be due to the blocking of catalytic active sites of Fe_3_O_4_ by excess rGO layers together with the decreased amount of Fe_3_O_4_ in the nanocomposites. Thus, it is clear that an optimum amount of rGO is compulsory to get a good conversion with high product selectivity.

The high catalytic performance of the rGO/Fe_3_O_4_ nanocomposites for the cyclohexene oxidation could be related to the synergistic effects of Fe_3_O_4_ nanoparticles with rGO in the composites. A catalytic oxidation reaction is usually accompanied by a redox reaction.^69^ In the present case it is expected that the Fe_3_O_4_ undergoes a redox cycle such as Fe^2+^ to Fe^3+^ (oxidation) and Fe^3+^ to Fe^2+^ (reduction) during the reaction and the transfer of electron could be facilitated by the presence of rGO.^70^ The rGO enhances the transfer of electrons compared to GO due to the presence of a well-developed conjugated network of π electrons in it, originated by the partial reduction of surface groups in GO. Therefore, the active catalyst sites were generated rapidly in each redox cycle for further catalytic reaction. A plausible reaction mechanism and the active species involved in reaction was studied separately at the end of the article.

**Fig. 9:**
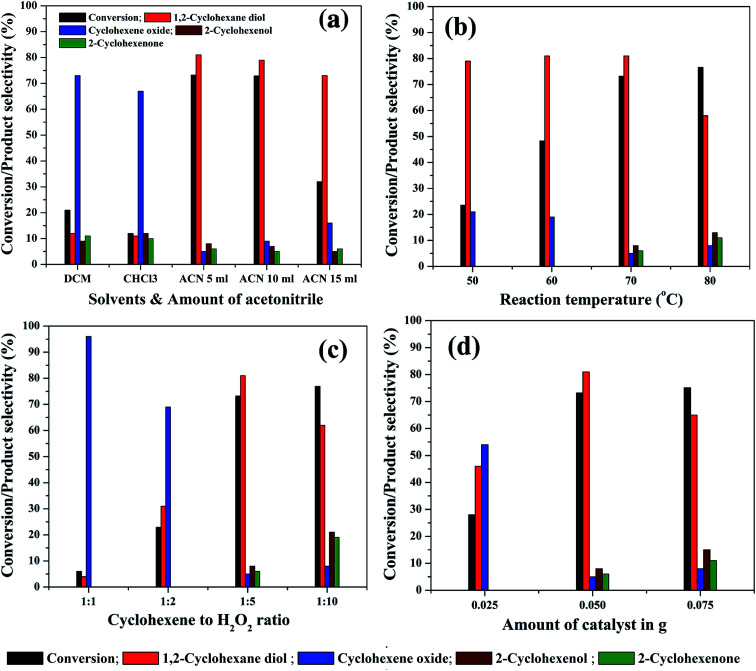
Effect of various reaction parameters: (a) solvents and its amount [cyclohexene: 2 mmol, H_2_O_2_: 10 mmol, solvent: 5 ml, catalyst: 5% rGO/Fe_3_O_4_ nanocomposite, catalyst dosage: 0.05 g, temperature: 70 °C, reaction time: 5 hours, atmospheric pressure], (b) effect of reaction temperature [cyclohexene: 2 mmol, H_2_O_2_: 10 mmol, acetonitrile: 5 ml, catalyst: 5% rGO/Fe_3_O_4_ nanocomposite, catalyst dosage: 0.05 g, reaction time: 5 hours, atmospheric pressure], (c) effect of cyclohexene/H_2_O_2_ molar ratio [cyclohexene: 2 mmol, acetonitrile: 5 ml, catalyst: 5% rGO/Fe_3_O_4_ nanocomposite, catalyst dosage: 0.05 g, temperature: 70 °C, reaction time: 5 hours, atmospheric pressure], (d) effect of catalyst dosage [cyclohexene: 2 mmol, H_2_O_2_: 10 mmol, acetonitrile: 5 ml, catalyst: 5% rGO/Fe_3_O_4_ nanocomposite, temperature: 70 °C, reaction time: 5 hours, atmospheric pressure].

In order to study the effect of reaction parameters on cyclohexene conversion and products selectivity, the parameters were varied individually by keeping the other parameters constant in the cyclohexene oxidation. The 5% rGO/Fe_3_O_4_ nanocomposite was used for the optimization studies since it gave maximum cyclohexene conversion and diol selectivity. At first, the effect of solvent was studied since it plays a significant role in the reaction and product selectivity. No conversion was observed in the solvent free liquid phase oxidation of cyclohexene. When water was used as solvent, nearly 10.7% cyclohexene conversion with 47.3% epoxide and 51.6% diol was formed without any allylic oxidation products. The low efficiency of cyclohexene oxidation in water could be attributed to the insolubility of cyclohexene in water and also due to its protic nature. It is known that the aprotic solvents increase the substrate concentration around the active catalytic sites more effectively than protic solvents. Therefore, three aprotic solvents such as acetonitrile, dichloromethane and chloroform were selected for cyclohexene oxidation. As shown in Fig. 9(a), the maximum cyclohexene conversion and diol selectivity was observed when the reaction carried out in acetonitrile solvent.^71^ Only 21% and 12% cyclohexene conversion with diol selectivities of 12% and 11% was observed when dichloromethane and chloroform was used as the solvents. Cyclohexene oxide was the main product with these solvents. The high conversion of cyclohexene in acetonitrile solvent could be due to its high dipole moment.^72^ Since it is polar aprotic solvent, it is expected to increase the substrate concentration to favour the reaction kinetics and hence cyclohexene conversion could be increased. The amount of acetonitrile was further increased to 10 ml and 15 ml to investigate its effect on cyclohexene conversion and products selectivity. It can be seen that no appreciable change was noticed in the cyclohexene conversion for 10 ml solvent whereas the conversion decreased to 32% when the amount of acetonitrile increased to 15 ml. However, the selectivity towards diol remained almost same in all amounts of acetonitrile. The low cyclohexene conversion in high volume of solvent could be due to the less contact time of the substrate with active catalyst sites because of the reduced substrate concentration in the reaction mixture. Therefore 5 ml of acetonitrile was selected for the remaining studies.

Reaction temperature is the other important factor which determines the efficiency of a liquid phase redox reaction. At room temperature, nearly 8.25% cyclohexene conversion with high epoxide selectivity (∼95%) was observed. Therefore, the effect of reaction temperature in the range of 50 °C to 80 °C was further studied for cyclohexene oxidation and the results are shown in Fig. 9(b). The cyclohexene conversion was found to be significantly increased from 23.5% to 73.2% with increasing the reaction temperatures from 50° to 70 °C. The selectivity to diol remained more or less same (80 ± 1%) in all of these temperatures. Furthermore, no allylic oxidation products were observed at 50 °C and 60 °C whereas cyclohexene oxide was detected as other side product with a selectivity of 20 ± 1% at these temperatures. The formation of allylic oxidation products was found to be starting from 70 °C onwards. When the reaction temperature increased to 80 °C, the cyclohexene conversion was slightly increased to 76.6% with a low diol selectivity of 58%. The oxidant H_2_O_2_ was added very carefully, without its decomposition. This is highly desired at this temperature. The selectivity of cyclohexene oxide, enol and enone was calculated to be 8%, 13% and 11% respectively at 80 °C. Thus, it is seen that the high reaction temperature increases the selectivity towards the allylic oxidation products rather than epoxidation product. However, our result is slightly deviated from the results of Yang *et al.*,^73^ possibly due to the difference in the catalysts. They reported that at low temperatures, the reaction proceeds through the allylic oxidation pathway resulting in the selective formation of α,β unsaturated alcohol and ketone whereas at high temperatures of 70–80 °C, the reaction resulted in the formation of more epoxide products such as epoxide and diol over TiO_2_ modified V_2_O_5_/MoO_3_ catalysts. However, the formation of allylic oxidation products could be due to the radical polymerization/chain mechanism as reported by Zhong *et al.* and Dou *et al.*^74,75^ Furthermore, the reaction was not conducted at 90 °C, since 82 °C is the reflux temperature of acetonitrile and the further increase of reaction temperature might lead to the rapid decomposition of H_2_O_2_ without taking part in the reaction. The high conversion of cyclohexene with increasing reaction temperatures could be due to the increased reaction rate according to Arrhenius theory of temperature dependent reaction rates. By considering the highest diol selectivity, 70 °C was selected for further optimization studies, without giving priority to the maximum conversion of cyclohexene at 80 °C.

The effect of substrate to oxidant molar ratio was studied by changing the amount of H_2_O_2_ in the liquid phase cyclohexene oxidation. The amount of H_2_O_2_ was varied from 2 mmol to 20 mmol whereas the other reaction parameters were kept constant. As shown in Fig. 9(c), the cyclohexene conversion drastically increased from 6% to 73.2% with an increase in the molar ratio of cyclohexene to H_2_O_2_ from 1 : 1 to 1 : 5. However, when the ratio increased to 1 : 10, only a small increment in cyclohexene conversion was noticed, which is not so significant and promising compared to the results of low substrate to oxidant ratios. The cyclohexene conversion was measured to be 6%, 22.9%, 73.2% and 76.9% respectively for the 1 : 1, 1 : 2, 1 : 5 and 1 : 10 cyclohexene to H_2_O_2_ molar ratios. The high conversion of cyclohexene with increasing H_2_O_2_ concentration could be due to the availability of more oxidant species for the substrate molecules at the active catalyst interfaces. However, the selectivities of the reaction products were found to be completely depending on the oxidant concentration. At low molar ratios of cyclohexene to H_2_O_2_, *i.e.* for 1 : 1 and 1 : 2 molar ratios, the main product was cyclohexene oxide, with highest selectivities of 96% and 69% respectively. The remaining contained only diol and no allylic oxidation products were detected. The effect of long duration of reaction time was further investigated in cyclohexene oxidation with a low substrate to H_2_O_2_ ratio of 1 : 2 under same experimental conditions. It indicated that there is no increase in cyclohexene conversion for a period of 10 h of reaction without any significant change in the product selectivities. Moreover, no allylic oxidation products were formed even after a long duration of reaction.

When the oxidant ratio increased to 1 : 5, surprisingly, the conversion reached to 73.2% with a maximum diol selectivity of 81%. Moreover, in addition to epoxide, allylic oxidation products also formed in small quantities. The further increase of oxidant concentration (1 : 10) has an adverse effect on the diol selectivity. The diol selectivity was dropped to 62% and the concentration of allylic oxidation products increased significantly. The selectivity of cyclohexene oxide, cyclohexenol and cyclohexenone was calculated to be 8%, 21% and 19% respectively. This indicates that the excessive use of H_2_O_2_ does not favour the selective oxidation of cyclohexene into diol whereas the selective formation of allylic oxidation products was initiated. Therefore, 1 : 5 molar ratio of cyclohexene to H_2_O_2_ was selected as the optimum ratio for the selective liquid phase oxidation of cyclohexene to diol.

The amount of catalyst was varied from 25 mg to 75 mg to study its effect on cyclohexene conversion and products selectivity. As seen in Fig. 9(d), the cyclohexene conversion increased with increasing the catalyst weight as expected, due to the availability of more active catalytic sites in the reaction mixture. At a low catalyst weight of 25 mg, only epoxidation products were observed with 46% diol and 54% epoxide selectivity with a cyclohexene conversion of 28%. The further increment of catalyst weight to 50 mg increased the cyclohexene conversion to 73.2% with 81% diol selectivity. A small increase in cyclohexene conversion (75.1%) was observed when the reaction conducted with 75 mg catalyst. However, the selectivity of diol decreased to 65% with the increased formation of allylic oxidation products. Therefore, 50 mg of catalyst was found to be optimum for getting high cyclohexene conversion with maximum diol selectivity in the present experimental conditions.

**Fig. 10:**
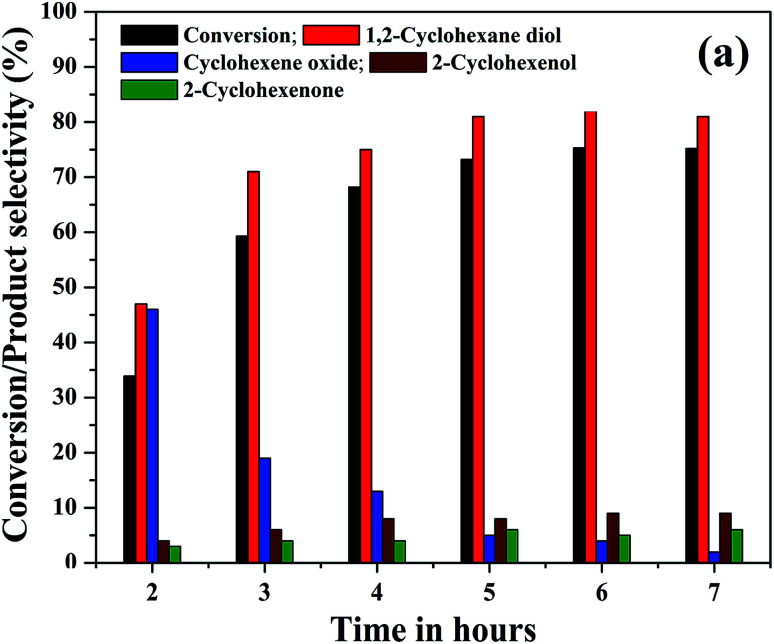
(a) Effect of reaction time on the cyclohexene conversion and products selectivity (cyclohexene: 2 mmol, H_2_O_2_: 10 mmol, acetonitrile: 5 ml, catalyst: 5% rGO/Fe_3_O_4_ nanocomposite, catalyst dosage: 0.05 g, temperature: 70 °C, atmospheric pressure).

The effect of reaction time on cyclohexene conversion and product selectivities was studied in the liquid phase oxidation of cyclohexene and the results are shown in Fig. 10. The cyclohexene conversion was found to be gradually increased with reaction time. As shown in the figure, the cyclohexene conversion increased from 34% to 73.2% as the time increased from 2 to 5 hours. Further increase of reaction time by one hour only increased the conversion to 75.3%. After 6 hours of reaction, no significant change was noticed in the cyclohexene oxidation, because the calculated cyclohexene conversion for 7^th^ hour remained same as that measured in the 6^th^ hours of reaction. Moreover, it is found that the selectivity of cyclohexene epoxide decreased gradually with time. At the same time a gradual increase in the diol selectivity was observed along with the formation of allylic oxidation products. A highest diol selectivity of 81% was obtained at 5^th^ hour of the reaction and it remained unchanged for further prolonged duration of reaction. The increased diol selectivity with reaction time could be due to the hydrolysis of the formed epoxide.

Fig. S3† shows the effect of different oxidants in the cyclohexene conversion and selectivity of the products. As seen, the H_2_O_2_ assisted reaction achieved a maximum cyclohexene conversion of 73.2% with 81% diol selectivity. However, the use of *tert*-butyl hydroperoxide (TBHP) as an oxidant in the reaction increased the substrate conversion to 82% with a highest epoxide selectivity of 77%. The diol selectivity was found to be only 13% in this case. It indicates the role of different oxidants in the selective distribution of the products.

A number of mechanisms were previously reported for cyclohexene oxidation depending on the product distribution, type of oxidants and catalysts used for the reaction. Based on the selective formation of diol, a plausible reaction mechanism was proposed. The oxidation of cyclohexene follows different pathways to achieve the products as reported. In the first pathway, the direct epoxidation of cyclohexene occurs and yields cyclohexene oxide. It further undergoes hydrolysis to produce 1,2-cyclohexene diol. In the second pathway, the oxidation of cyclohexene occurs at allylic position and results in the formation of 2-cyclohexenol. It further oxidizes to 2-cyclohexenone. Since 1,2-cyclohexane diol was the major product formed in all of the reactions along with the formation of direct epoxidation and allylic oxidation products, a different mechanism is expected together with the first mechanistic pathway (hydrolysis of epoxide), which is not the same, as reported in many of the articles. Therefore, at first, a radical scavenger study was performed using butylated hydroxytoluene as a radical scavenger. The results had shown that the addition of 5 mmol of butylated hydroxytoluene to the reaction mixture drastically decreased the cyclohexene conversion to 9% with a lowest diol selectivity of 2.1%. Cyclohexene epoxide and allylic oxidation products such as cyclohexenol and cyclohexenone were formed with the selectivities of 2.8%, 41.1% and 54% respectively. The decreased cyclohexene conversion confirms that the epoxidation proceeds through a radical mechanism. Previously Zubir *et al.*^76^ reported the formation of radical species by H_2_O_2_ decomposition over GO/Fe_3_O_4_ composite. According to them, a synergistic interfacial effect of Fe_3_O_4_ and graphene oxide was observed in the composite for the facile formation of radical species through a Fenton like mechanism *via* electron transfer.

Initially, the oxidant H_2_O_2_ gets adsorbed on the solid liquid interface of the nanocomposite and the active catalytic sites of Fe_3_O_4_ (Fe^2+^ and Fe^3+^), anchored on the surface of reduced graphene oxide decomposes the adsorbed H_2_O_2_ into hydroxyl (˙OH) and hydroperoxyl (˙OOH) radicals respectively. At this stage, Fe^2+^ and Fe^3+^ ions inter convert each other by their redox reaction as per eqn (1) and (2). In addition to Fe_3_O_4_, the rGO also takes part in this process. This is mainly due to the intrinsic donor–acceptor surface characteristics of carbon nanomaterials.^77^ The adsorbed oxidant H_2_O_2_ further get activated on the surface of rGO and decomposes to hydroxyl and hydroperoxyl radicals according to eqn (3) and (4). Herein, the sp^2^ (C=C) and sp^3^ (C–C) carbon atoms present in the rGO act as the reduced and oxidized carbon active sites respectively. Therefore, more radical species were formed in the reaction mixture according to eqn (1)–(4).^75^

1 Fe^2+^ + H_2_O_2_ → Fe^3+^ + ˙OH + OH^−^

2 Fe^3+^ + H_2_O_2_ → Fe^2+^ + ˙OOH + H^+^

3 sp^2^ rGO_C=C_ + H_2_O_2_ → sp^3^ rGO_C–C_ + ˙OH + OH^−^

4 sp^3^ rGO_C–C_ + H_2_O_2_ → sp^2^ rGO_C=C_ + ˙OOH + H^+^

**Fig. 11:**
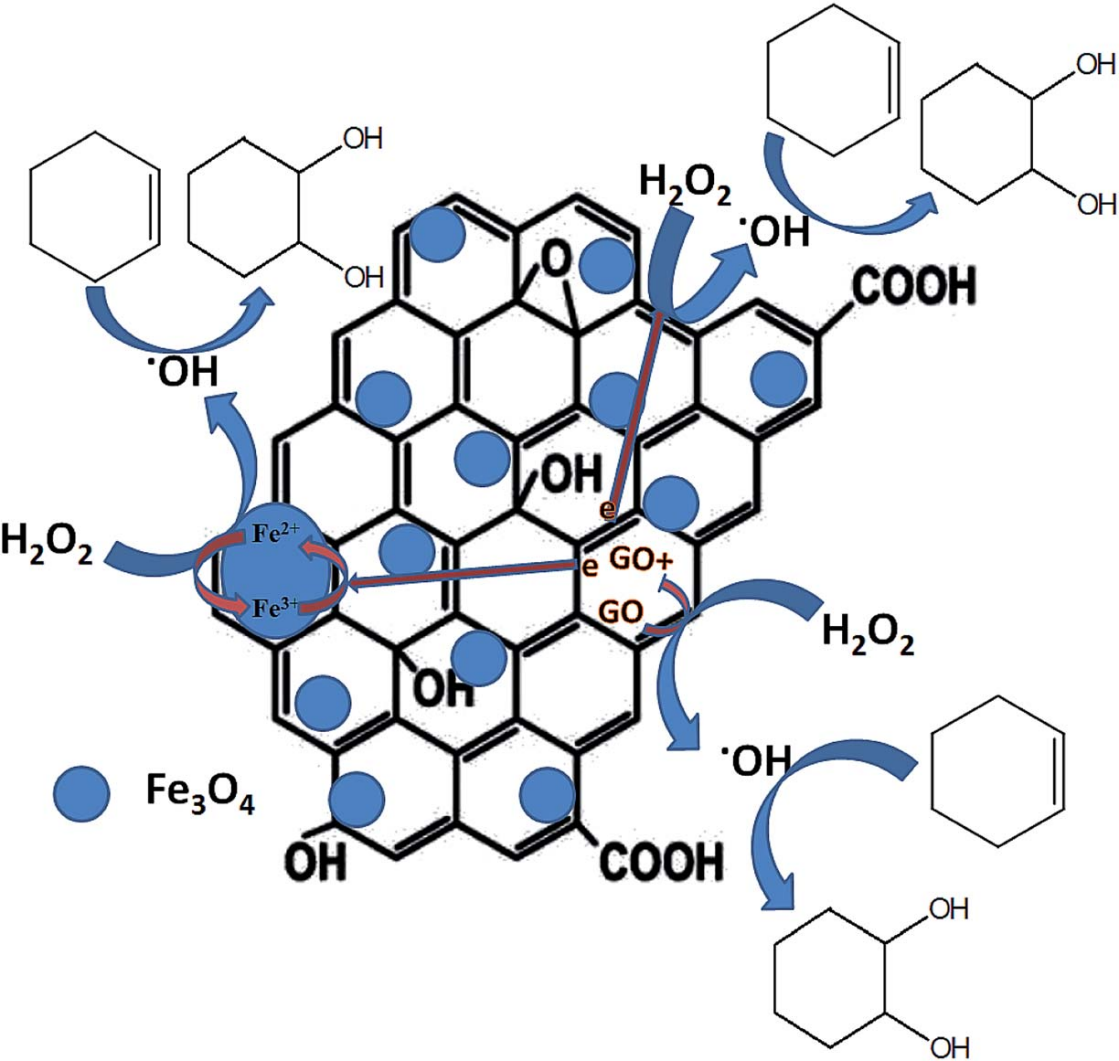
Schematic representation of the plausible mechanism for cyclohexene oxidation.

Since reduced graphene oxide contains a conjugated network of π electrons, there is a good path for the rapid transfer of electrons between rGO and active iron sites. The presence of Fe–O–C bondings in the rGO/Fe_3_O_4_ nanocomposite further facilitates the transfer of electrons in the composite with an excellent reduction potential of graphene oxide.^76^ Thus, the redox cycle of the active centers in Fe_3_O_4_ could be enhanced, due to the synergistic effects of rGO and Fe_3_O_4_ nanoparticles. Or more clearly it can be said that the redox cycle of Fe^2+^/Fe^3+^ system could be achieved quickly due of the presence of conjugated network of π electrons in rGO. In addition to this, the sacrificial role of reduced graphene oxide for transferring electron to Fe_3_O_4_ by the oxidation of its sp^2^ carbon domains is significant. This further explains the effectiveness of rGO/Fe_3_O_4_ nanocomposites for cyclohexene oxidation compared to their individual components. The formed radicals especially hydroxyl radicals further react with cyclohexene on the catalysts surface and the selective formation of diol with high cyclohexene conversion could be achieved. It is reported that the hydroxyl radicals are more reactive than the hydroperoxyl radicals since their reaction rate constants are very low compared to hydroxyl radicals.^78^ Therefore, it is expected that the hydroxyl radicals play a major role in the oxidation of cyclohexene. A schematic representation of plausible mechanism is shown in Fig. 11.

**Fig. 12:**
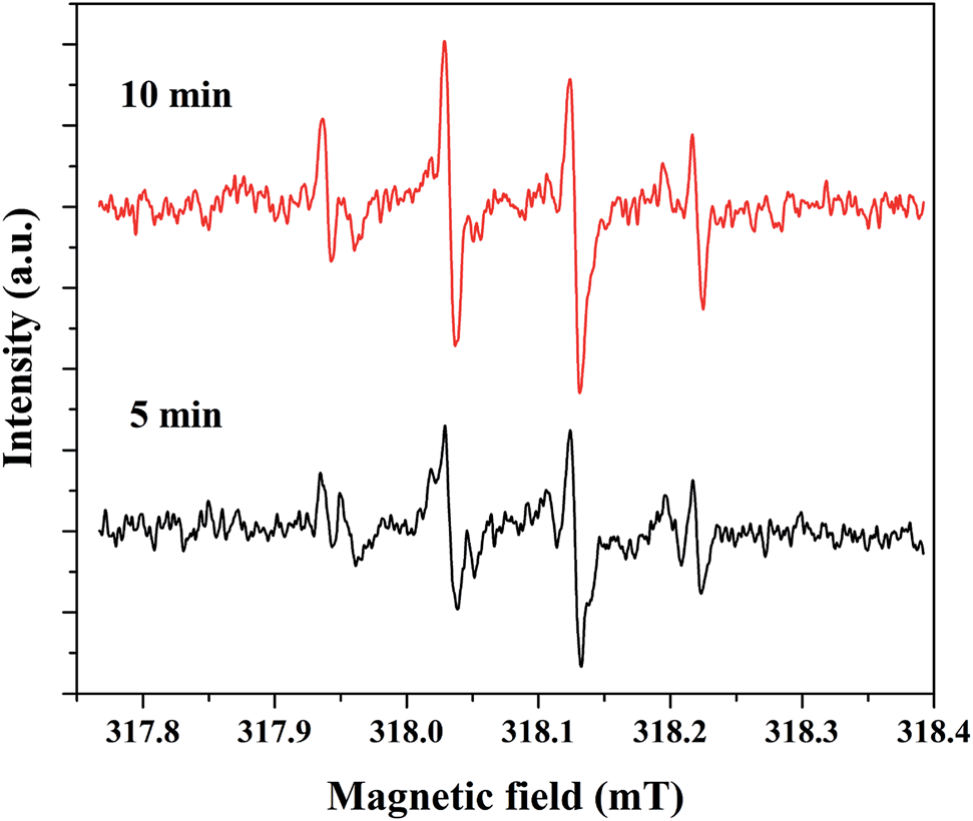
DMPO-˙OH profiles for 5% rGO/Fe_3_O_4_ nanocomposite measured by ESR analysis.

The electron spin resonance (ESR) spectroscopic method was applied to confirm the formation of hydroxyl radicals. For this study, 5% rGO/Fe_3_O_4_ composite was selected as a representative sample and the spectrum was recorded using 5,5-dimethyl-1-pyrroline-*N*-oxide (DMPO) as a radical trapping agent in the presence of H_2_O_2_. The resulting spectrum at two different time intervals of 5 and 10 minutes is shown in Fig. 12. It showed the presence of four peaks in the form of DMPO-˙OH signals with a peak intensity ratio of 1 : 2:2 : 1, which confirms the formation of hydroxyl radicals.

**Fig. 13:**
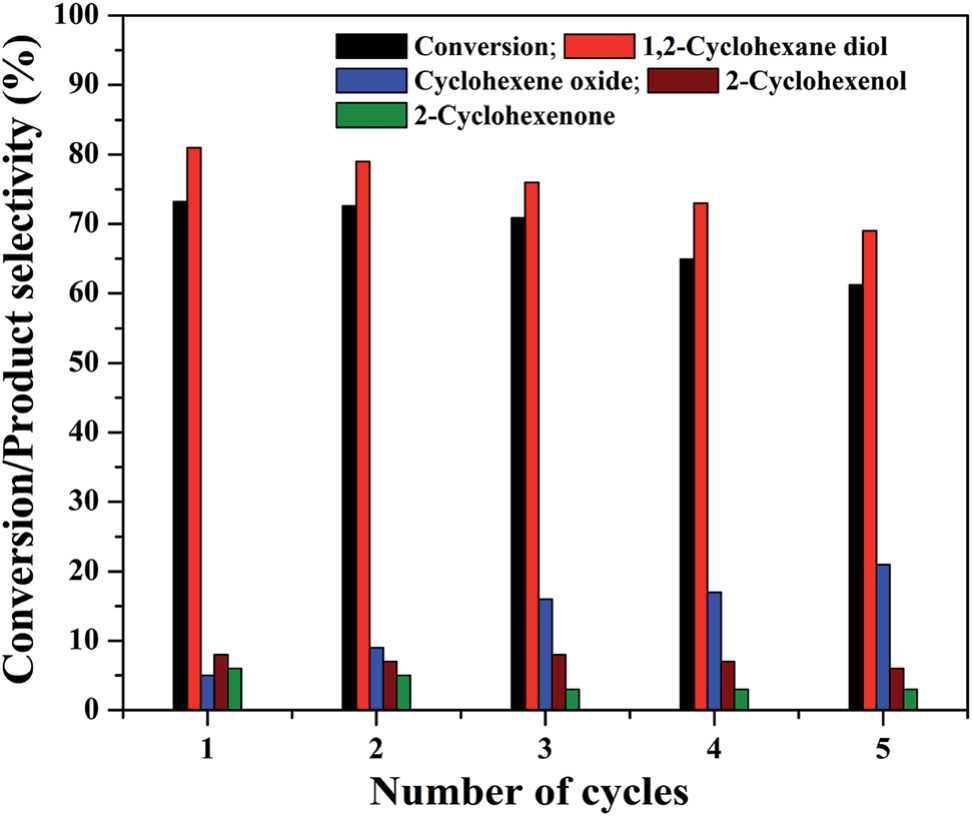
Reusability of the 5% rGO/Fe_3_O_4_ nanocomposite for cyclohexene oxidation.

The practical application of a heterogeneous catalyst mainly depends on its reusability for successive run in the reaction. Therefore, four reusability studies were conducted after separating the catalyst from reaction mixture using an external magnet. The magnetically separated catalysts were washed with deionized water followed by ethanol and dried at 75 °C before its use for the next run. The same experimental conditions were used for the reusability studies and the results are shown in Fig. 13. It can be seen that the cyclohexene conversion and diol selectivity decrease slightly in each run with a simultaneous increase in the epoxide selectivity. The cyclohexene conversion was found to be 61.2% for the fourth reusability study with a diol selectivity of 69%. It indicates that there is a gradual activity loss in the continuous reaction. It could be attributed to the leaching of metal species in the nanocomposite during its continuous use. It was further confirmed by metal leaching test measured by ICP-OES analysis. A part of the reaction mixture obtained in the first and fourth reusability run, after the separation of catalyst was analyzed by ICP-OES to check the possibility of any iron content in the solution. The results show that there is a small amount of iron loss due to metal leaching was observed during the reusability studies. It was measured to be less than 2% (1.8 ± 0.2%) in both cases. This metal leaching could be responsible for the decreased performance of the catalyst for the continuous reaction.^79^ The reduction in the reaction efficiency for continual run is not so much significant while concerning the run number. As the catalyst is active for the continuous reaction, even though there is a slow decrease in its performance; it is worth to mention their stability.

**Fig. 14:**
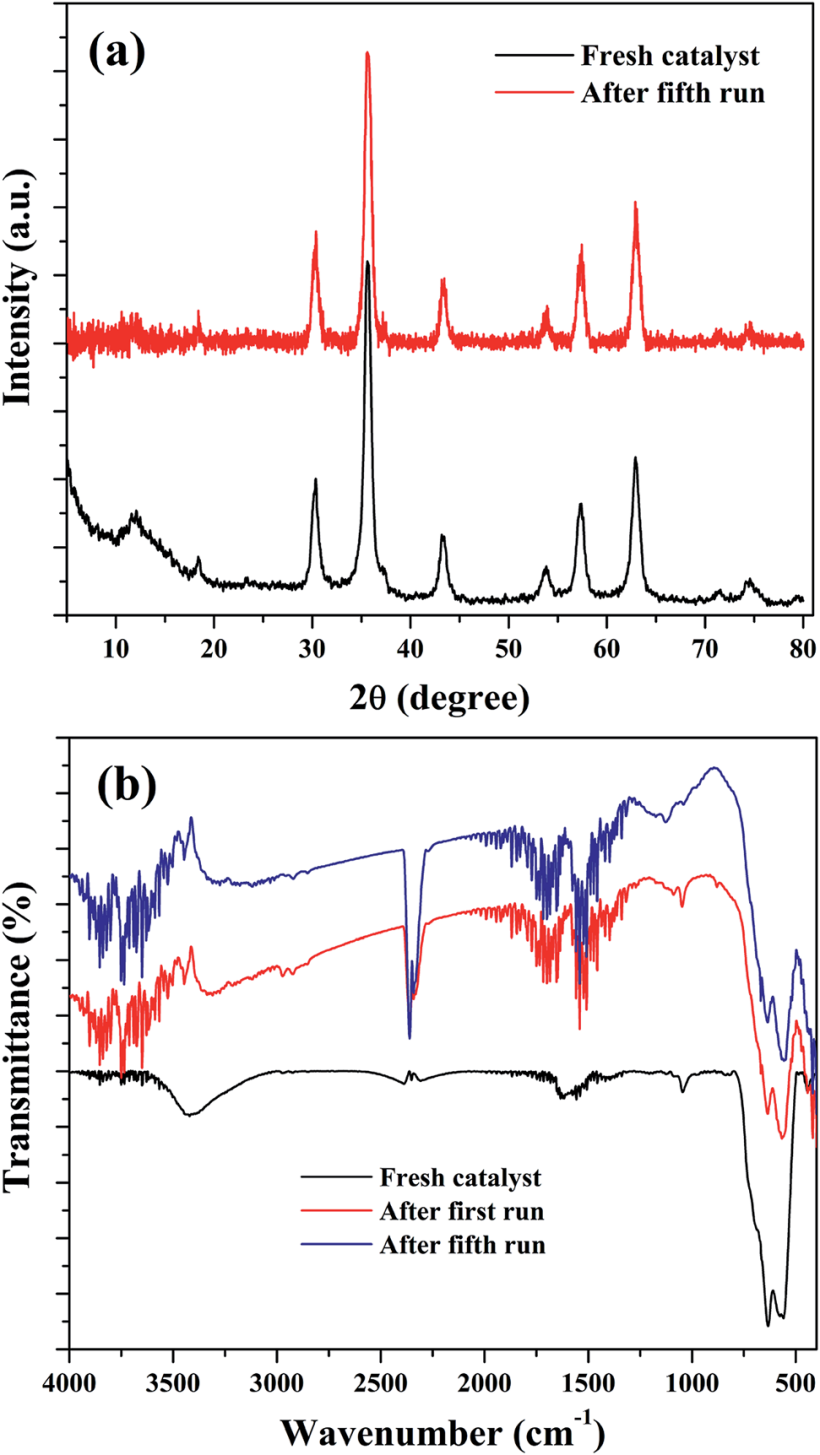
(a) XRD and (b) FTIR spectra of the reused samples.

The stability of the nanocomposites for continuous reaction could be due to the maintenance of its structural features. Therefore, XRD and FTIR analysis were used to characterize the reused catalysts for their crystalline and structural properties. As shown in Fig. 14(a), there is no significant crystalline change was observed. The peaks of Fe_3_O_4_ were found to be retained in the reused catalysts with a slight reduction in the intensity of peak at 35.6°. This could be attributed to the metal leaching process. Similar observation was also noted in the FTIR results. Fig. 14(b) shows the FTIR spectra of the reused catalysts and it indicate that the transmission bands related to Fe_3_O_4_ in the nanocomposite was not changed in the spent catalysts, obtained after the first and fourth reusability run of reaction. Moreover, some addition transmission bands were observed. These peaks could be originated from the oxidation of aromatic C=C bonds present in the rGO of nanocomposite in the H_2_O_2_ environment.^39,76^ The transmission bands observed at 1122 cm^−1^ and 1187 cm^−1^ could validate this assumption since these bands were related to oxygenated groups in graphene oxide. The intense transmission band at 2363 cm^−1^ could be originated from the adsorbed CO_2_, probably on the surface of graphene oxide. The changes in the surface texture of the reduced graphene oxide as a result of the continuous run could slow down the electron transfer through the disturbed conjugated network of π electrons in rGO to accomplish a redox cycle of the active catalyst sites of Fe_3_O_4_. This could be another possible reason for the decreased activity of the composite for continuous cyclohexene oxidation.

## 4. Conclusions

An ultrasonication assisted precipitation combining solvothermal method was reported to prepare a set of rGO/Fe_3_O_4_ nanocomposites with various amounts of graphene oxide and were successfully used for the selective liquid phase oxidation of cyclohexene to 1,2-cyclohexane diol using H_2_O_2_ as a green oxidant. The prepared nanocomposites were characterized for their crystalline, structural, textural and magnetic properties. The active phase of iron was found to exist in the form of magnetite in the nanocomposites. The field emission scanning and transmission electron microscopic analysis confirmed the fine surface dispersion of more or less spherical shaped Fe_3_O_4_ nanoparticles on the surface layers of rGO nanosheets. The Fe_3_O_4_ nanoparticles were found to be strongly anchored on the surface of rGO layers through Fe–O–C bonding, as indicated by the shift of binding energies of the representative elements in the XPS characterization. The magnetic studies indicated that the magnetic saturation values of Fe_3_O_4_ decreased by the incorporation of rGO and its incremental amount in the nanocomposites. However, the values were more than enough for its separation from the solution with the use of an external magnet. The increased of amount of rGO in the nanocomposites further enhanced the rate of particle dispersion and decreased the aggregation rate of Fe_3_O_4_ nanoparticles. The nitrogen physisorption analysis showed the enhancement of textural properties size as specific surface area and pore parameters after the formation of nanocomposites. Among the nanocomposites, 5% rGO/Fe_3_O_4_ composite showed highest activity and selectivity for cyclohexene oxidation. The low and high amounts of graphene oxide in the nanocomposite does not positively impacted the conversion and product selectivity.

The study on the effect of reaction parameters proven that a maximum cyclohexene conversion of 75.3% with a highest diol selectivity of 81% was achieved with a substrate to oxidant ratio of 1 : 5 using 0.05 g of 5% rGO/Fe_3_O_4_ catalyst at 70 °C in 5 ml of acetonitrile solvent for 6 h of reaction. The enhanced activity of the catalysts could be attributed to the synergistic effects of Fe_3_O_4_ nanoparticles that were effectively dispersed on the surface of rGO layers in the nanocomposites. The scavenger studies on reaction indicated the active role of hydroxyl radicals in the oxidation reaction, which could be formed by the decomposition of H_2_O_2_ by a Fenton type mechanism in Fe^2+^/Fe^3+^ system *via* electron transfer in the nanocomposites. Furthermore, due to the presence of a conjugated network of π electrons in rGO, the Fe^2+^/Fe^3+^ redox cycle could be fastened in the nanocomposites for the generation of more hydroxyl radicals for oxidation. The additional sacrificial role of rGO in the electron transfer process is more effective for the fast accomplishment of Fe^2+^/Fe^3+^ redox cycle. Moreover, the composites were successfully reused for four times in cyclohexene oxidation without any drastic loss in the activity.

## Conflicts of interest

There are no conflicts of interests to declare.

## Supplementary Material

RA-009-C9RA04685B-s001
