# Supplementary material for: Magnetically separable and reusable rGO/Fe3O4 nanocomposites for the selective liquid phase oxidation of cyclohexene to 1,2-cyclohexane diol
Source: RSC Adv. 2019 Oct 11;9(56):32517–34. doi: 10.1039/c9ra04685b (PMC9072983; doi:10.1039/c9ra04685b)
Supplement: RA-009-C9RA04685B-s001 [file RA-009-C9RA04685B-s001.pdf]

Electronic Supplementary Material (ESI) for RSC Advances.
This journal is © The Royal Society of Chemistry 2019

SUPPLEMENTARY DATA

Magnetically separable and reusable rGO/Fe_3O_4 nanocomposites for the selective liquid phase oxidation of cyclohexene to 1,2-cyclohexane diol

Manoj Pudukudy *, Qingming Jia *, Yanan Dong, Zhongxiao Yue, Shaoyun Shan
Faculty of Chemical Engineering, Kunming University of Science and Technology, Kunming, 650500, Yunnan, People's Republic of China
*Email: manojpudukudy@gmail.com; jiaqm411@163.com

Supplementary Figures

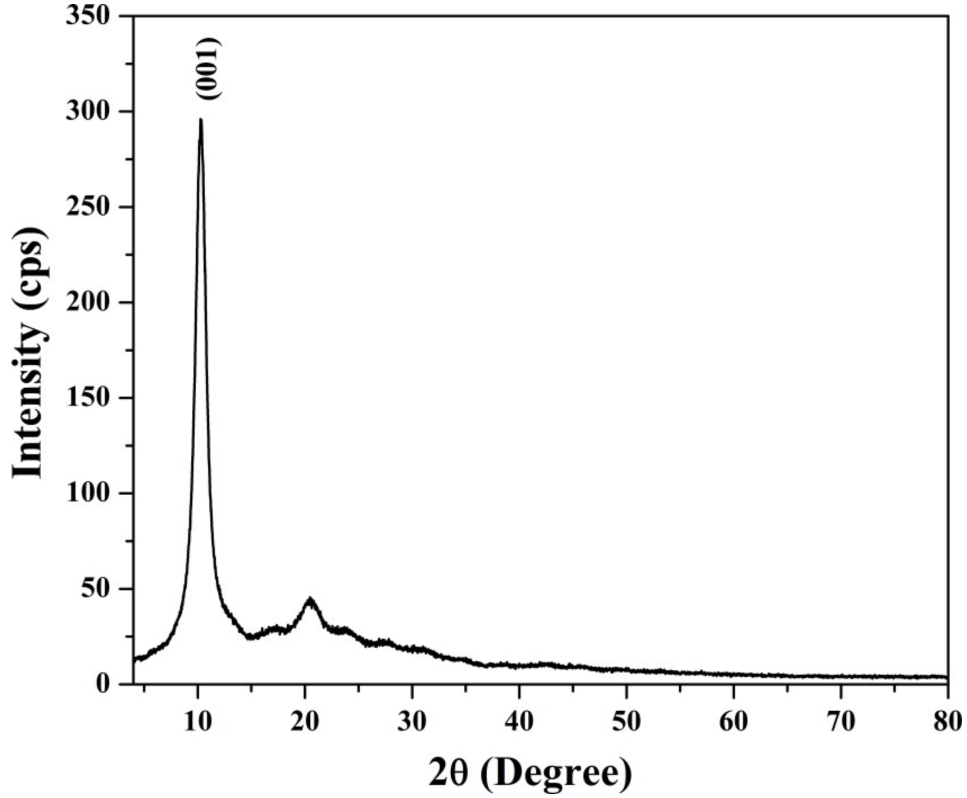

Fig. S1- XRD pattern of the graphite oxide prepared by modified Hummer's method

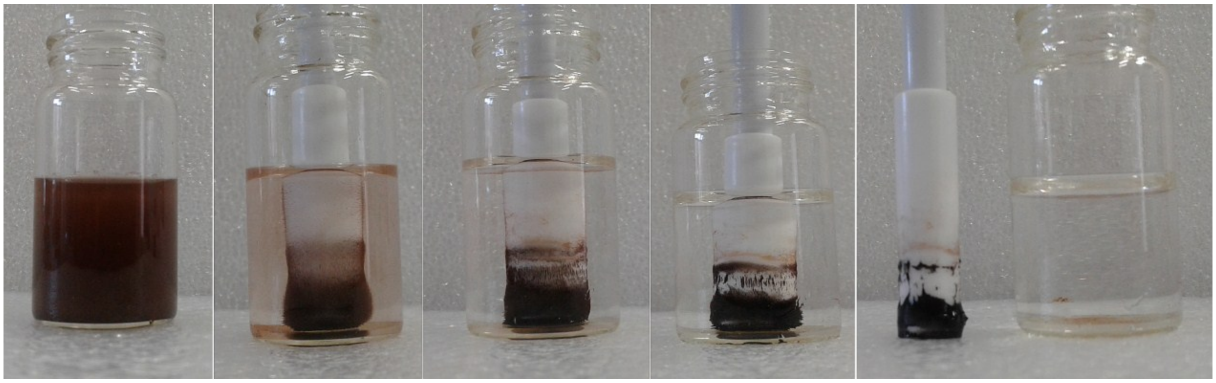

Fig. S2- Photograph of the magnetic separation of the nanocomposites from reaction mixture

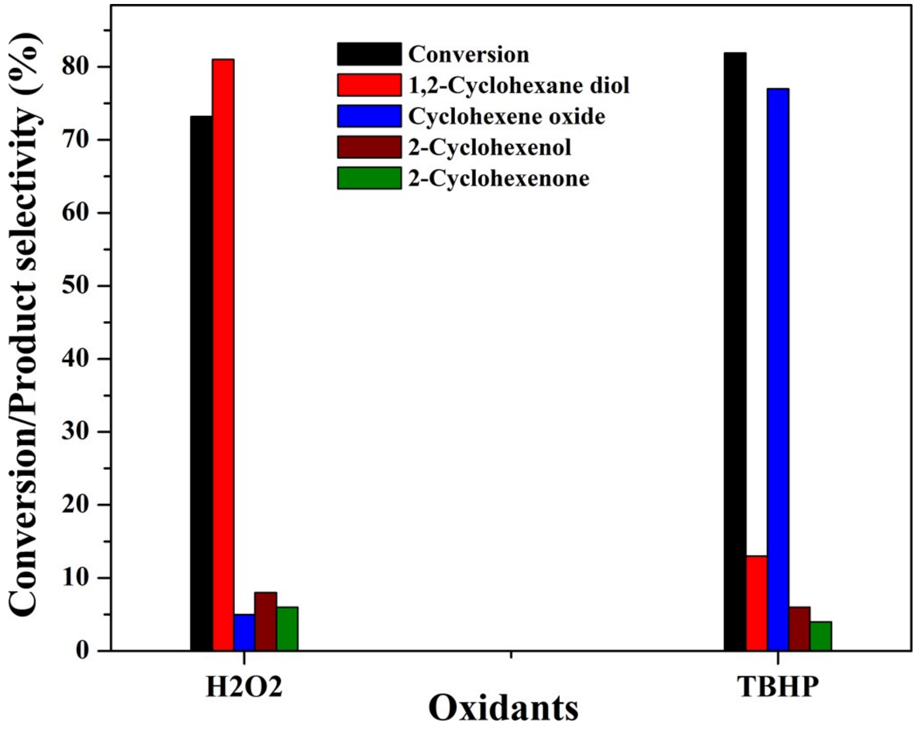

Fig. S3- Effect of different oxidants in the reaction (*Cyclohexene: 2 mmol,* H_2O_2*: 10 mmol, TBHP: 10 mmol; Acetonitrile: 5 ml, Catalyst: 5% rGO/*Fe_3O_4 *nanocomposite, Catalyst dosage: 0.05 g, Temperature: 70*^oC*, Reaction time:5 hours, atmospheric pressure*)
